# Disrupted synaptic vacuolar H^+^-ATPase renders synaptic vulnerability to Alzheimer’s disease

**DOI:** 10.1172/jci.insight.202983

**Published:** 2026-10-08

**Authors:** Yanting Chen, Khloud Ashraf Farouk Emam, Shuwen Yue, Jing Tian, Tienju Wang, Albert Park, Shalini Mishra, Gagan Deep, Zi-Jun Wang, Heng Du, Lan Guo

**Affiliations:** 1 Department of Neuroscience, Wake Forest School of Medicine, Winston-Salem, North Carolina, USA.; 2 Department of Pharmacology and Toxicology, University of Kansas, Lawrence, Kansas, USA.; 3 Alzheimer’s Disease Research Center and; 4 Department of Internal Medicine, Section on Gerontology and Geriatric Medicine, Wake Forest School of Medicine, Winston-Salem, North Carolina, USA.

**Keywords:** Aging, Neuroscience, Alzheimer disease, Neurodegeneration

## Abstract

Defective synaptic transmission is a prominent pathology that underlies cognitive deficits in Alzheimer’s disease (AD), highlighting the need to elucidate molecular mechanisms of synaptic failure. Vacuolar H+-ATPase (V-ATPase), a proton-pumping enzyme, is essential for synaptic vesicle acidification and neurotransmitter loading. However, whether SV-associated V-ATPase is vulnerable to AD remains unclear. Here, using SV-rich fractions from postmortem brain tissues, we identified SV-associated V-ATPase deficits, including decreased enzymatic activity, impaired complex assembly, and altered expression of its key subunits in AD. SV-associated V-ATPase dysfunction was further associated with pathological and clinical characteristics of AD. Genetic downregulation of the V-ATPase V1D subunit, a component reduced in AD brains, disrupted V-ATPase proton transport and impaired SV acidification. Further experiments using 5×FAD mice, which exhibited AD-like SV-associated V-ATPase deficits, demonstrated the deleterious impact of V-ATPase dysfunction on SV acidification and synaptic transmission including presynaptic neurotransmitter release. In addition, ex vivo studies identified amyloid β–induced oxidative stress as a driver of V1D loss and V-ATPase disassembly, linking AD pathology to SV-associated V-ATPase dysfunction. These findings indicate that SV-associated V-ATPase dysfunction contributes to synaptic failure and cognitive deficits in AD. Therapeutic avenues to mitigate V-ATPase dysfunction have the potential to attenuate synaptic failure for the management of AD.

## Abstract 2

<style type=”text/css”>a { text-decoration: none; color: #464feb; } tr th, tr td { border: 1px solid #e6e6e6; } tr th { background-color: #f5f5f5; } </style> Defective synaptic vesicle acidification impairs neurotransmission in Alzheimer’s disease, highlighting vacuolar H+-ATPase as a promising therapeutic target.

## Introduction

Alzheimer’s disease (AD) is an age-related neurodegenerative disorder clinically characterized by progressive memory loss and pathologically defined by synaptic degeneration (1). A strong clinicopathological correlation between synaptic dysfunction and cognitive deficits supports the classification of AD as a synaptopathy and highlights the role of dysregulated synaptic transmission in its pathophysiology (1–3). Although AD is typically not considered a neurotransmitter disorder, disturbances in a variety of neurotransmitters, such as acetylcholine and glutamate, are prominent in the brain in AD, offering opportunities for the development of symptom-modifying therapies (4, 5). Consistent with the notion that synaptic vesicles (SVs) are vehicles for neurotransmitters that subsequently control neurotransmitter release (6), clinical and basic research have reported SV pathologies including alterations in SV architecture and recycling and loss of presynaptic proteins involved in SV docking and neurotransmitter release in AD-related paradigms (7–10). These findings accentuate SV vulnerability in AD and further underscore the need to understand the mechanistic pathways behind SV deficits in this neurodegenerative disorder.

Vacuolar H^+^-ATPase (V-ATPase) is an evolutionarily conserved proton-pumping enzyme that is composed of 2 major domains including the transmembrane V0 and cytosolic V1 sectors (11–13). The V1 domain, which faces the cytosol, catalyzes ATP hydrolysis by rotating the V-ATPase central stalk, thereby initiating proton transport that is then completed by the exit of protons through the V0 domain (14, 15). This proton-pumping ability allows V-ATPase to create a proton gradient for the acidification of the lumens of various intracellular organelles, such as endosomes, lysosomes, and neuron-specific SVs, enabling them to fulfill their functions (13, 16–18). Echoing its functional importance to neurobiology, V-ATPase has been intensively investigated in AD-relevant pathological settings. Previous studies have associated V-ATPase abnormalities with lysosomal dysfunction in neurons and microglia, resulting in impaired neuronal function and dysregulated neuroinflammatory response, in various models of AD-related pathologies (19–23). Of note, current investigations of V-ATPase in AD have predominantly focused on lysosomal function, and the functional status of V-ATPase in SVs remains largely understudied. A recent study integrating multiomics analyses with experimental validation has linked V-ATPase dysfunction to impaired synaptic strength in AD-related contexts (24). These findings further highlight the importance of V-ATPase in synaptic function and underscore the need for a more detailed examination of SV-associated V-ATPase in the development of synaptic deficits in AD.

Here, we conducted a comprehensive study of SV-associated V-ATPase in postmortem AD brains and in a mouse model of AD-like amyloidosis, the 5×FAD mouse. We observed impaired V-ATPase proton transport and compromised complex integrity in AD-related conditions. Genetic knockdown of the V-ATPase V1D subunit, which was found to be downregulated in AD brains, reproduced the deleterious effects of the pharmaceutical blockade of V-ATPase proton transport on SV acidification in neurons. These results recapitulate the SV-associated V-ATPase and accompanying SV abnormalities seen in AD-related conditions. Electrophysiological studies provided further confirmation that V-ATPase proton transport dysfunction is associated with presynaptic deficits in relevant AD pathologies. Our results suggest that SV-associated V-ATPase dysfunction, particularly disrupted proton flow, is a phenotypic pathology that mediates synaptic failure in AD-related conditions. These findings advance our understanding of synaptic injury in AD pathogenesis and highlight the potential of V-ATPase-based therapeutic interventions for AD management.

## Results

### V-ATPase dysfunction in SV-rich fractions from patients with AD.

To determine the functional status of V-ATPase in patients with AD, we used postmortem temporal lobe tissues from 7 controls without AD and 7 age- and sex-matched patients (Supplemental Table 1; supplemental material available online with this article; https://doi.org/10.1172/jci.insight.202983DS1). The temporal lobe was selected for this study due to its cognitive importance and vulnerability to AD (25). We purified synaptosomes for SV-rich fractions by centrifugation (26) for further measurement of V-ATPase activity. Biochemical assays for ATP hydrolysis were conducted using SV-rich fractions in the presence of oligomycin A, ouabain, and thapsigargin, which are selective inhibitors of F-type, Na^+^/K^+^-, and Ca²^+^-ATPases, respectively (27–29). Assays were conducted with and without concanamycin A, a selective inhibitor that blocks V-ATPase proton transport via the V0 domain (30), to determine the activity of functionally assembled V-ATPase. Analysis of the data showed that V-ATPase in SV-rich fractions from patients with AD exhibited reduced concanamycin A–sensitive capacity to hydrolyze ATP as compared with their counterparts from controls without AD (Figure 1A). In addition, we also measured V-ATPase activity in brain homogenates and observed a decrease in AD samples compared with controls (Supplemental Figure 1A). However, further analysis revealed no significant difference in the magnitude of V-ATPase reduction between brain homogenates and SV-rich fractions in AD (Supplemental Figure 1B). These findings suggest that V-ATPase dysfunction in SVs may occur in the context of a global reduction in V-ATPase activity, rather than representing a selectively compartment-specific defect within the examined brain region at this disease stage.

Because the inhibitory effect of concanamycin A is reliant on the integrity of the V-ATPase complex (30), we next subjected the SV-rich fractions to the examination of V-ATPase complex assembly by blue native PAGE (BN-PAGE) (20). By immunoblotting using a specific antibody recognizing V0c, a key subunit of the V0 domain (14), we observed a reduction in fully assembled complexes alongside an elevation of assembly intermediates containing the V0c subunit (Figure 1, B and C), indicating disrupted V-ATPase complex assembly in SV-rich fractions from patients with AD. Of note, to achieve structural stability and functional efficiency, the V-ATPase complex has a defined and regulated subunit stoichiometry (18). Therefore, disrupted complex integrity further prompted us to examine the expression of key subunits of V-ATPase. Immunoblotting using SV-rich fractions from individuals with and without AD was performed to detect the key subunits of V-ATPase, including V1A1, V1B2, V1C1, V1D, V1E1, and V1F in the V1 domain and V0a1, V0c, and V0d1 in the V0 domain. Among the subunits examined, densitometric analysis showed a selective reduction in the V1D subunit in samples from patients with AD (Figure 1, D and E). V1F levels were also significantly decreased in AD SVs, although the magnitude of reduction was less pronounced compared with V1D (Figure 1, D and E). Given that both V1D and V1F are structurally coupled components of the central stalk of V-ATPase (31), these findings thus align with disrupted V-ATPase assembly in patients with AD. Taken together, our results support V-ATPase dysfunction in presynaptic terminals, which may affect SV function, in patients with AD.

### Clinicopathological relationship of SV-associated V-ATPase dysfunction with cognitive deficits in AD.

To determine the clinical relevance of SV-associated V-ATPase dysfunction to AD, we performed a receiver operating characteristic (ROC) analysis. By using SV-associated V-ATPase activity and AD diagnosis as covariables, our ROC analysis showed an AUC of 0.8980 (Figure 2A; *P* = 0.0127), suggesting the potential to use the functional status of SV-associated V-ATPase to distinguish patients with AD from control individuals. Echoing the ROC analysis, linear regression analysis determined a positive relationship between SV-associated V-ATPase activity and cognitive performance, which was determined by the proxy of using patient Mini-Mental State Examination (MMSE) scores (Figure 2B), indicating an association between SV-associated V-ATPase dysfunction and cognitive deficits in AD. To correlate SV-associated V-ATPase dysfunction with AD-related pathologies, we extended our observations and determined a positive relationship between V-ATPase activity and brain weight (Figure 2C). Further analysis showed an inverse relationship of V-ATPase activity with brain amyloidosis (Figure 2D) and fibrillary tangles (Figure 2E). These findings collectively suggest that SV-associated V-ATPase dysfunction is a phenotypic pathology associated with dementia. They also add credence to the hypothesis that this dysfunction contributes to the synaptic failure and resultant cognitive deficits seen in AD.

### Dysfunction of V-ATPase in SV-rich fractions from 5×FAD mice.

V-ATPase’s proton-pumping function, which requires complex integrity and ATP-hydrolyzing capacity, is fundamental to its regulation of SVs (32). It is therefore important to examine whether V-ATPase dysfunction underlies impaired SV acidification in AD-related conditions. Being aware of the technical difficulty of measuring SV acidification in postmortem human tissues, we therefore employed 5×FAD mice, a mouse model of AD-like brain pathology, in our study (33). SV-rich fractions were purified from the synaptosomes of age- and sex-matched nontransgenic (non-Tg) and 5×FAD mice at 8–10 months old, when 5×FAD mice exhibit prominent brain amyloid β (Aβ) accumulation, synaptic transmission deficits, and cognitive impairment (34). A V-ATPase enzymatic activity assay using SV-rich fractions showed decreased V-ATPase activity, demonstrated by lowered ATP hydrolysis capacity, in 5×FAD mice compared with their non-Tg counterparts (Figure 3A). Recapitulating findings from human samples, V-ATPase activity was also reduced in whole-brain homogenates from 5×FAD mice (Supplemental Figure 1C) with no difference in the magnitude of reduction observed between brain homogenates and SV-rich fractions (Supplemental Figure 1D). Consistent with reduced enzymatic activity, further BN-PAGE using SV-rich fractions showed a decrease in the number of full complexes alongside an increase in assembly intermediates determined by anti-V0c immunoblotting (Figure 3, B and C). Next, we subjected the samples to immunoblotting for the major V-ATPase subunits, including V1A1, V1B2, V1C1, V1D, V1E1, and V1F and V0a1, V0c, and V0d1. We found that 5×FAD mice exhibited a specific reduced expression of V1D in their SV-rich fractions (Figure 4, A and B), corroborating the findings from patients. Regardless of the discrepancy in V-ATPase V1F subunit vulnerabilities, 5×FAD mice shared key features with patients with AD, including impaired activity and disrupted complex integrity of SV-associated V-ATPase, thus validating the use of this model to study the impact of V-ATPase dysfunction on SV acidification in AD-relevant settings.

SV-rich fractions were purified from non-Tg and 5×FAD mice at 8–10 months old, and fresh samples were subjected to SV acidification assays using acridine orange, a membrane-permeable fluorescent dye, as an indicator of proton gradients (20, 35). To confirm the purity of the SV preparation, freshly isolated fractions were examined by transmission electron microscopy, which verified the enrichment of morphologically intact vesicles (Supplemental Figure 2A). This was further supported by immunoblot analysis of synaptophysin and synaptotagmin, as well as cathepsin B and cathepsin D, which serve as SVs and lysosomal markers, respectively (36, 37) (Supplemental Figure 2B). Because all experimental groups were processed in parallel using identical isolation procedures, potential technical variability in vesicle integrity or size distribution was minimized.

At the emission wavelength of 520 nm, SV-rich samples from both non-Tg and 5×FAD mice in the absence of ATP demonstrated a steady line with no or little fluctuation in fluorescence (Figure 4C). This stable baseline fluorescence reflects initial membrane integrity of the purified SVs across groups, ruling out generalized vesicle disruption or severe leakage prior to stimulation. The addition of ATP triggered a fluorescence decrease in samples from both groups, which was not seen in the presence of Bafilomycin A1 (BafA1), a specific inhibitor of V-ATPase proton flow (Figure 4C) (38), which, in combination with no fluorescence change in the absence of ATP, indicated activated V-ATPase–driven proton transport into SVs. However, in contrast to their counterparts from non-Tg mice, the decreasing rate of ATP-triggered change in acridine orange fluorescence was attenuated in SVs from 5×FAD mice (Figure 4C), suggesting attenuated ATP-driven SV acidification in this mouse model of AD-like pathology. Although our findings of a blunted response of SVs from 5×FAD mice to ATP-stimulated changes in acridine orange fluorescence implicate a contribution of V-ATPase dysfunction, it should also be noted that a sustained proton gradient in SVs is maintained by voltage-gated chloride channels (CLCNs, also referred to in literature as ClCs), especially CLCN3 through its function in neutralizing the intravesicular positive charge to facilitate continuous proton buildup (39). However, our examination of SV-rich fractions by immunoblotting showed an increase in CLCN3 expression (Supplemental Figure 3, A and B). In this regard, the data together support the association between V-ATPase dysfunction and SV acidification impairment in AD-related conditions.

### Impact of AD-associated V1D deficiency on SV proton transport in vitro.

Next, we extended our observations to establish a direct link between AD-associated V-ATPase changes and SV acidification defects. As shown in Figure 1E and Figure 4B, we determined that loss of V1D is a shared V-ATPase change between patients with AD and 5×FAD mice. Given the importance of V1D for V-ATPase structural stability (18, 40), we hypothesized that loss of V1D per se is able to impair SV acidification. To this end, we genetically knocked down V1D in neuronal cultures by using lenti-shRNA-V1D. Knockdown efficiency was verified at both the transcriptional and translational levels. Quantitative real-time PCR and immunoblotting demonstrated a reduction in *Atp6v1d* mRNA (Figure 5A) and protein (Figure 5B) expression, respectively, compared with nontargeting scrambled shRNA controls. Further examination by biochemical assays for V-ATPase activity and BN-PAGE using an antibody against V0c recapitulated V-ATPase dysfunction, including lowered ATP hydrolysis capacity (Figure 5C) and enhanced V-ATPase disassembly (Figure 5, D and E) as observed in patients and 5×FAD mice. Upon these observations, we purified SV-rich fractions from neuronal cultures and measured SV acidification, reflected by changes in acridine orange fluorescence in the presence of ATP. As expected, V1D-downregulated neurons demonstrated impaired ATP-driven SV acidification (Figure 5F), indicating the deleterious impact of V-ATPase dysfunction on the buildup of a proton gradient in SVs. Lastly, loss of V1D and resultant V-ATPase dysfunction reduced synaptic density in cultured neurons (Supplemental Figure 5), corroborating the previously reported effect of long-term SV dysfunction in suppressing synaptogenesis and synapse formation (41). These results suggest that AD-related changes in V-ATPase, at least in the loss of V1D, are sufficient to cause synaptic dysfunction, even in the absence of AD-associated toxic molecules. This supports the hypothesis that SV-associated V-ATPase dysfunction may contribute to synaptic transmission impairment in AD.

### Impact of disrupted V-ATPase proton transport on synaptic transmission in 5×FAD mice.

To determine whether V-ATPase dysfunction is involved in synaptic transmission defects in AD-related conditions, we subjected organotypic brain slices from non-Tg and 5×FAD mice at 8–10 months old to bath application of vehicle or BafA1, a specific inhibitor of V-ATPase proton flow (38). Pyramidal neurons in the neocortex were selected for the recording of spontaneous excitatory postsynaptic currents (sEPSCs). In the vehicle-treated groups, neurons from 5×FAD mice demonstrated a decrease in both the frequency (Figure 6, A and C) and amplitude (Figure 6, B and C) of sEPSCs compared with their non-Tg counterparts. The data are in agreement with our previous observations (42), indicating impaired synaptic transmission that covers both presynaptic and postsynaptic components in 5×FAD mice. In the BafA1-treated groups, neurons from non-Tg mice exhibited a robust response to the inhibition of V-ATPase proton transport, reflected by a reduction in sEPSC frequency (Figure 6, A and C) and amplitude (Figure 6, B and C). However, such synaptic response to BafA1 was diminished in 5×FAD neurons (Figure 6, A–C). Furthermore, to reflect SV function, we examined SV release probability through evoked presynaptic glutamate release as measured by the paired-pulse ratio (PPR) of EPSCs. EPSC PPR was elevated in 5×FAD pyramidal neurons compared with controls (Figure 6, D and E), suggesting a decreased probability of glutamate release from the presynapse. Additionally, BafA1 bath application caused an increase in the PPR of EPSCs in pyramidal neurons from non-Tg mice (Figure 6, D and E), indicating the sensitivity of non-Tg neurons to BafA1-mediated PPR presynaptic suppression and the importance of V-ATPase function to presynaptic transmission. In contrast, pyramidal neurons in 5×FAD mice were unresponsive to BafA1 challenges in the PPR assay (Figure 6, D and E). These results collectively support decreased flexibility of V-ATPase–modulated synaptic transmission in 5×FAD mice, which is possibly due to a floor effect of V-ATPase dysfunction. These observations further suggest the contribution of impaired V-ATPase proton transport to synaptic failure in AD-relevant pathological settings.

### Effect of Aβ-induced oxidative stress on SV-associated V-ATPase function ex vivo.

The overlapping deficits in SV-associated V-ATPase observed in both patients with AD and the Aβ-based mouse model support a potential role for Aβ toxicity in promoting SV-associated V-ATPase dysfunction in AD-related conditions, prompting us to further investigate the underlying mechanisms. Notably, oxidative stress has been linked to disrupted V-ATPase function and complex integrity (43), and increased oxidative stress, driven by excess ROS, is a well-established downstream consequence of Aβ toxicity that contributes to cellular dysfunction and neurodegeneration (44, 45). In this context, we examined the effects of Aβ exposure and oxidative stress modulation using SV-rich fractions derived from Aβ-treated organotypic brain slice cultures. Specifically, SV-rich fractions were isolated from brain slices incubated with or without Aβ, in the presence or absence of pretreatment with N-acetylcysteine (NAC), a potent ROS scavenger (46). SDS-PAGE immunoblot analysis demonstrated a selective reduction in the V1D subunit (Figure 7A) among the major V-ATPase subunits (Supplemental Figure 6, A and B) after Aβ exposure, which was accompanied by dissipated V-ATPase assembly determined by BN-PAGE (Figure 7B). In contrast, such deleterious effects of Aβ on V-ATPase were attenuated by NAC in a dose-dependent manner (Figure 7 and Supplemental Figure 6). Collectively, these findings demonstrate SV-associated V-ATPase vulnerability to Aβ toxicity, potentially via ROS-dependent destabilization of the V1D subunit and consequent impairment of complex assembly.

## Discussion

Neurons actively transmit information to each other, and these interneuronal communications through synaptic transmission are fundamental to learning and memory (47). Impaired synaptic transmission is shown to compromise cognitive performance and has been linked to cognitive disorders including AD (2, 48). Synaptic transmission is a tightly controlled process, beginning with the presynaptic loading of neurotransmitters into SVs, followed by activity-triggered neurotransmitter release from SVs, and ending with the postsynaptic response to those neurotransmitters (49). Previous clinical and basic research has highlighted postsynaptic changes with a special emphasis on the deregulation of postsynaptic receptors such as the NMDA receptor (50) and the α-amino-3-hydroxy-5-methyl-4-isoxazolepropionic acid (AMPA) receptor (51) in the pathogenesis of synaptic failure in AD dementia. However, although presynaptic damage is determined in AD (7–10), the precise mechanisms rendering the vulnerability of presynapses, especially SVs, have not yet been elucidated in this neurodegenerative disorder. In this study, we observed changes in V-ATPase including key subunit loss, structural instability, and proton-pumping incapacity in SV-rich fractions from patients with AD. Further clinical association studies showed a correlation of V-ATPase dysfunction with decreased brain weight and impaired cognitive performance, highlighting a possible contribution of V-ATPase dysfunction in presynaptic terminals, due to its direct regulation of SV recycling (52), to the pathogenesis of AD.

In our clinical association study, we observed a positive correlation between V-ATPase dysfunction and both brain amyloidosis and neurofibrillary tangle burden, indicating a close association between V-ATPase impairment and AD pathologies. Consistent with this observation, we detected V-ATPase deficits in 5×FAD mice and in Aβ-treated brain slices, which model conditions of elevated Aβ burden. In addition, prior studies have reported deleterious effects of amyloid precursor protein β-C-terminal fragment (APP β-CTF), Aβ, and hyperphosphorylated tau on V-ATPase function (20, 23). Together, these findings support the opinion that V-ATPase dysfunction arises, at least in part, as a downstream consequence of AD-associated pathological processes. At the same time, emerging evidence indicates that lysosomal V-ATPase dysfunction can contribute to Aβ pathology by impairing endolysosomal acidification and APP processing (19–23), suggesting an additional upstream role of V-ATPase dysfunction in AD. In this context, V-ATPase dysfunction may participate in a feedforward pathogenic cycle with AD pathology, particularly Aβ, in which each process reinforces the other during disease progression. Notably, SV-associated and lysosomal V-ATPase pools reside in distinct cellular compartments and serve different physiological functions, with lysosomal dysfunction primarily affecting proteostasis and Aβ metabolism (19–23), whereas SV-associated dysfunction directly affects neurotransmitter loading and synaptic transmission. Because APP processing and Aβ generation predominantly occur within the endosomal-lysosomal system rather than in SVs (53, 54), this feedforward interaction between V-ATPase dysfunction and Aβ pathology is likely driven predominantly by lysosomal V-ATPase impairment. Nevertheless, despite this compartmental distinction in Aβ pathology, these pathway-specific alterations may still converge at the level of synaptic integrity, where impaired proteostasis and disrupted neurotransmission act in concert to drive synaptic failure in AD. Because of current technical limitations that preclude selective manipulation of SV-associated V-ATPase in isolation, direct experimental evaluation of its contribution to Aβ or tau pathology, as well as synapto-pathology, remains challenging. Nonetheless, our findings, together with prior literature, support a model in which SV-associated V-ATPase dysfunction contributes to synaptic impairment during disease progression, and future studies employing refined compartment-specific approaches will be essential to define the temporal and causal relationships between V-ATPase dysfunction and AD pathogenesis.

Moreover, in this study, we also noticed compromised complex stability of V-ATPase in AD-related conditions, which agrees with a previous report (20) and raises great interest in the molecular basis of increased V-ATPase disassembly accompanying AD. This is particularly important because the proton-pumping function of V-ATPase is reliant on its complex integrity (52). Through immunoblot analyses, we determined a reduction of the V1D and V1F subunits in SV-associated V-ATPase from patients with AD. Given the established roles of these subunits in facilitating V-ATPase assembly (18, 40), together with our findings from neurons deficient in the V1D subunit, these results suggest that loss of these V1/V0 interface components contributes to compromised V-ATPase integrity at presynaptic sites. We further identified Aβ-associated oxidative stress as a pathological driver promoting V-ATPase disassembly in ex vivo settings. Consistent with this finding, antioxidant treatment rescued these alterations, supporting a role for Aβ-induced ROS in mediating V1D subunit loss and destabilization of the V-ATPase complex. These findings underscore the contribution of oxidative stress to V-ATPase dysfunction in AD-relevant settings and corroborate a prior report linking oxidative stress to disrupted V-ATPase complex integrity (43). Nevertheless, additional mechanisms that contribute to V-ATPase impairment in AD cannot be ignored. For example, phosphorylated APP β-CTF is reported to bind V0 subunits to inhibit V-ATPase complex assembly (20). In addition, transcriptomic and multiomics studies have identified reduced *V1A* gene expression (24, 55) and highlighted its role as a key regulator of neuronal gene networks disrupted in AD (24). However, we did not detect changes in V1A protein levels in our patient samples. This discrepancy may reflect differences across cohorts, dissociation between mRNA and protein expression, or variation in subcellular fractions analyzed, particularly given our focus on SV preparations. Interestingly, aside from V1D, SV-associated V-ATPase exhibited differences in subunit vulnerability between patients with AD and 5×FAD mice. This discrepancy may reflect limitations of the 5×FAD model, which primarily recapitulates Aβ pathology without robust tau involvement (33), as well as the shorter lifespan of rodents, which constrains modeling of age-dependent processes. It should be noted that lysosomal V-ATPase is influenced by pathological tau (23), in which case, it is also possible that the interactions between Aβ, pathological tau, and other aging factors synergistically contribute to V-ATPase dysfunction with the development of AD. Collectively, these outstanding questions highlight the complexity of molecular pathways underlying V-ATPase dysfunction and emphasize the need for further mechanistic studies, which may ultimately inform the development of more effective V-ATPase–targeted therapeutic strategies for AD.

Lastly, V-ATPase constantly undergoes reversible disassembly as a physiological mechanism to regulate its energy-consuming proton-pumping function (20). Under normal conditions, the balance between assembly and disassembly is tightly controlled and responsive to cellular metabolic states. Functionally, such disassembly is likely to exert stage-dependent effects on SV biology. Previous studies suggest that non–V1-connected “free” V0 domains retain biological function in regulating neurotransmitter release from SVs, while reassembly of the V1/V0 complex is sensitive to reductions in the luminal proton gradient (52). In this study, we did not directly examine the contribution of impaired V1/V0 assembly to SV release in patients or 5×FAD mice. However, the suppressed SV release probability and diminished responsiveness to BafA1 challenges in 5×FAD mice indicate that impaired neurotransmitter SV refill due to V-ATPase disassembly is likely to overshadow any potential release-promoting effects from augmented “free” V0. In addition, it is suggested that “free” V0 only acts on neurotransmitter-filled SVs (52). Therefore, in a thought experiment, it is possible that V-ATPase dysfunction, upon its initial occurrence, not only dissipates SV refill due to compromised SV acidification but also promotes unleashed neurotransmitter release from prefilled SVs due to an increase in “free” V0. This provides a possible mechanism behind both the initial neuronal hyperactivity and the subsequent synaptic failure throughout AD progression (56).

In this proof-of-concept study, we linked V-ATPase dysfunction to synaptic transmission defects in AD-related conditions. Our findings not only provide insight into underlying mechanistic pathways but also suggest that V-ATPase dysfunction could be a therapeutic target for AD. However, several outstanding questions arise from our study, including the synergistic effect of lysosomal and SV V-ATPase dysfunction on synaptic function in AD and the dynamic changes of V-ATPase with AD progression, which will be addressed in our future in-depth study. The most parsimonious interpretation of our results is that SV-associated V-ATPase dysfunction is a pathological phenotype contributing to synaptic injury and cognitive deficits in AD. Therefore, therapeutic strategies that restore V-ATPase proton-pumping function hold promise for mitigating cognitive decline in patients with AD.

## Methods

### Sex as a biological variable.

Both male and female individual samples were included in this study. There were no statistically significant differences in the sex ratio between the groups, as shown in Supplemental Table 1. Both male and female mouse samples were included in this study. There were no statistically significant differences in the sex ratio between the groups (2-tailed Fisher’s exact test, *P* > 0.99).

### Human samples.

Frozen postmortem temporal lobe tissues were requested from the Brain and Body Donation Program at Banner Sun Health Research Institute and the University of Kansas Alzheimer’s Disease Research Center. Patients’ demographic and neuropathological data were collected by the institutions, and the study adhered to the Declaration of Helsinki principles.

### Mice.

All animal procedures were approved by the University of Kansas IACUC and Wake Forest University School of Medicine (WFUSM) IACUC and conducted in compliance with the NIH *Guide for the Care and Use of Laboratory Animals* (National Academies Press, 2011). The 5×FAD mice (B6SJL-Tg (APPSwFlLon, PSEN1*M146L*L286V) 6799Vas/Mmjax, The Jackson Laboratory, strain 034840) and B6SJLF1/J (The Jackson Laboratory, strain 100012) were maintained in the University of Kansas Animal Care Unit or WFUSM Animal Resources Program under standard housing conditions (12-hour light/12-hour dark cycle, controlled temperature and humidity). Breeding was performed in-house to generate experimental cohorts. No backcross was performed for the mouse strain. Genotypes of animals were confirmed using PCR and/or amyloid plaque staining. Sex- and age-matched 5×FAD and non-Tg mice were used in the study. The number of mice was determined by our previous data and power calculation to ensure that the minimal number of mice required were used in the experiments.

### Primary cultured neurons.

Primary neuron cultures were prepared as previously described (57, 58). In brief, mouse hippocampal tissues were dissected from postnatal day 0–1 pups and dissociated using 0.05% trypsin at 37°C for 15 minutes, followed by homogenization in ice-cold DMEM. Dissociated cells were then passed through a 100 μm cell strainer (Corning) and centrifuged for 5 minutes at 200*g*. The pellet was gently resuspended in neuron culture medium (Neurobasal A with 2% B27 supplement, 0.5 mM L-glutamine, Invitrogen) and plated on cell culture plates coated with poly-D-lysine (Sigma-Aldrich) or Lab-Tek chamber slides (Nunc) with appropriate densities. At 6 days in vitro (DIV), lentivirus carrying shRNA targeted to mouse *Atp6v1d* (3LVS(LVshRNA)-C-73834-1, Vector builder) or nontarget scramble shRNA (LVM(VB010000-0016nbs)-C, Vector builder) were applied to neurons at an MOI of 5. Cells were collected at 11–12 DIV for the experiments.

### SV-rich fraction preparation.

The mouse SV-rich fractions were extracted as previously described (59). Briefly, mice were anesthetized with isoflurane, and the cortex and hippocampus were dissected. Brains were homogenized with a Dounce homogenizer in 5 mL of ice-cold buffer A (320 mM sucrose, 10 mM HEPES, pH 7.4) supplemented with protease inhibitors (Roche). The homogenate was centrifuged at 1,300*g* for 15 minutes at 4°C. The supernatant (S1) was collected and centrifuged at 30,000*g* for 20 minutes at 4°C. The resulting pellet (P2) was resuspended and homogenized in ice-cold low-osmolarity buffer B (32 mM sucrose, 10 mM HEPES, pH 7.4) using a Dounce homogenizer. After incubation on ice for 45 minutes, the homogenate was again centrifuged at 30,000*g* for 20 minutes at 4°C. This supernatant was then collected and centrifuged at 160,000*g* for 2 hours at 4°C. The resulting pellet, identified as the crude SV-rich fractions, was resuspended in buffer A using a 23G needle for experiments. SV enrichment and purity were validated by transmission electron microscopy and immunoblot analysis of SV-specific and lysosomal markers.

Human synaptosomes were obtained from human temporal lobe tissues as previously described (26). The purified synaptosomes were permeabilized in 0.02% digitonin buffer and incubated on ice for 5 minutes. The resulting lysate was centrifuged at 20,000*g* for 20 minutes at 4°C to obtain the SV-rich supernatant for subsequent experiments.

Primary neurons cultured on 10 cm dishes were harvested in ice-cold PBS. The neurons were homogenized in 1 mL of cold buffer A with protease inhibitors using a Dounce homogenizer. After removing unbroken cells by centrifugation at 2,400*g* for 5 minutes, the homogenate was centrifuged at 14,000*g* for 10 minutes at 4°C to pellet synaptosomes. After resuspending in buffer B, synaptosomes were homogenized and kept on ice for 15 minutes before adding 0.2 mL buffer C (1.5 M sucrose, 10 mM HEPES, pH 7.4) to restore osmolarity. The final mixture was centrifuged at 15,000*g* for 10 minutes to clear unbroken synaptosomes and debris, and the resulting SV-rich fraction in the supernatant was stored at 4°C for subsequent experiments.

### Acidification assay.

ATP-dependent SV acidification was monitored by the quenching of acridine orange (Thermo Fisher Scientific). Next, 6 μg of SV-rich fraction was preincubated in 100 μL of assay buffer (150 mM sucrose, 10 mM MgCl_2_, 100 mM KCl, 1 μM acridine orange, 20 mM HEPES, pH 7.0). After a stable baseline was achieved, 2 mM Mg^2+^/ATP was added to initiate V-ATPase-mediated acidification. Control groups to isolate ATP hydrolysis and proton translocation were either subjected to the assay without the addition of 2 mM Mg^2+^/ATP or preincubated with BafA1 (10 μM), respectively. The changes in acridine orange fluorescence (excitation at 485 nm and emission at 528 nm) were monitored using a BioTek NEO2 microplate reader at 32°C, with data acquisition every 10 seconds. Fluorescence changes were expressed as the percentage decrease in acridine orange fluorescence relative to the first recorded value immediately after ATP addition, reflecting V-ATPase–dependent SV acidification.

### V-ATPase activity.

V-ATPase activity was quantified utilizing an NADH-coupled ATPase assay. SV-rich fractions were diluted in assay buffer (100 mM Tris, 2 mM MgCl_2_, 50 mM KCl, 0.2 mM EDTA, 0.23 mM NADH, 1 mM phosphoenolpyruvate, 36 μg/μL pyruvate kinase, pH 7.4). To ensure specific measurement of V-ATPase activity, the assay buffer was supplemented with 0.2 μM oligomycin A, 100 μM ouabain, and 0.5 μM thapsigargin to inhibit F-type, Na^+^/K^+^-, and Ca²^+^-ATPases, respectively, and assays were performed in the presence or absence of 200 nM concanamycin A. The reaction mixture was preincubated and allowed to equilibrate at 37°C for 5 minutes. The reaction was initiated by the addition of 0.20 mM Mg²^+^/ATP. Decreased NADH concentration via reduction to NAD^+^ was measured by decrease in absorbance at 340 nm using a BioTek NEO2 microplate reader. Measurements were taken for 120 seconds at 10-second intervals. The rate of NADH consumption was used to calculate ATPase activity, which was then normalized to total protein input in the reaction system. V-ATPase–specific activity was defined as the concanamycin A–sensitive fraction of ATP hydrolysis, calculated as the difference between total ATPase activity measured in the absence of concanamycin A and the residual (concanamycin A insensitive) activity measured in its presence, under conditions where the activities of other types of ATPase were pharmacologically inhibited. Because concanamycin A selectively inhibits the proton-translocating V0 domain of intact and functionally coupled V-ATPase complexes, the concanamycin A–sensitive component primarily reflects ATP hydrolysis mediated by functional V-ATPase. This approach minimizes contributions from residual non–V-ATPase activity as well as uncoupled or disrupted V-ATPase components present in the preparation.

### Native gel immunoblot analysis.

The SV-rich fractions were solubilized in cold 1% digitonin, incubated on ice for 30 minutes, and then centrifuged at 20,000*g* for 20 minutes at 4°C to remove insoluble material (58). The cleared supernatant was mixed with 4× NativePAGE sample buffer and 1% NativePAGE G-250 sample additive (Thermo Fisher Scientific, BN2008). The samples were then subjected to electrophoresis on NativePAGE Novex 4%–16% Bis-Tris gels using 1× NativePAGE Running buffer (Thermo Fisher Scientific, BN2007), followed by transfer to 0.45 μm PVDF membranes (Bio-Rad) for subsequent immunodetection.

### Gel electrophoresis and immunoblotting.

Samples were prepared in NuPAGE LDS sample buffer and incubated for 5 minutes at 100°C followed by electrophoresis on 10% Bolt Bis-Tris Plus Mini Protein gels (Thermo Fisher Scientific, NW00105BOX). Proteins were transferred to PVDF membranes (Bio-Rad, 1620177). Then, the membranes were blocked with 5% nonfat milk at room temperature for 1 hour and incubated with the primary antibodies overnight at 4°C (Supplemental Table 2). After probing secondary antibodies, blots were developed using SuperSignal West Pico PLUS Chemiluminescent Substrate (Thermo Fisher Scientific, 34580). or Atto Ultimate Sensitivity Substrate (Thermo Fisher Scientific, A38556). Images were captured and analyzed with a ChemiDoc XRS+ Gel Imaging System (Bio-Rad).

### Real-time PCR.

The primary cultured neurons were infected with lentivirus carrying shRNA targeted mouse *Atp6v1d* or nontarget scramble control shRNA at DIV 6 and harvested at DIV 11–12. Total RNA was extracted using RNeasy Mini kit (QIAGEN, 74104). cDNA was synthesized using Takara PrimeScript RT Master Mix (RR036B). mRNA expression was examined using the following primer pair: *Atp6v1d* forward 5′-GGCAAAGACCGGATTGAAATCT-3′ and reverse 5′-GTCGAAATCGAAGAGTTAAGGCA-3′ (PrimerBank 12963799a1, 130 bp); *Gapdh* forward 5′- AGGTCGGTGTGAACGGATTTG-3′ and reverse 5′- TGTAGACCATGTAGTTGAGGTCA -3′ (PrimerBank, 6679937a1, 123bp). Real-time PCR was performed and analyzed with QuantStudio 5 real-time PCR system (Thermo Fisher Scientific).

### Organotypic brain slice culture and treatment.

Organotypic brain slice cultures were prepared as previously described (60) with minor modifications. Briefly, after decapitation, whole brains from adult mice were rapidly removed under sterile conditions and placed in ice-cold, oxygenated (95% O_2_/5% CO_2_) sucrose-substituted Ringer’s solution. Brain regions containing cortex and hippocampus were sectioned into 350 μm thick slices using a vibratome (Leica, VT1200S). Slices were carefully transferred onto Millicell membrane inserts (MilliporeSigma) maintained at the interface of air and culture medium in a humidified incubator equilibrated with 5% CO_2_ at 37°C. For pharmacological treatment, brain slices were pretreated with NAC (500 μM or 1mM) for 2 hours, followed by exposure to oligomeric Aβ42 (500 nM) or vehicle control for 24 hours. After treatment, brain slices were harvested and processed for SV-rich fraction isolation using a protocol analogous to that applied for whole mouse brain tissue.

### Oligomeric Aβ preparation.

Oligomeric Aβ42 was prepared as previously described (34). Briefly, Aβ42 peptide (GenicBio, A-43-T-1000) was dissolved in 1,1,1,3,3,3-hexafluoro-2-propanol (Sigma-Aldrich) to 1 mM, aliquoted, and dried overnight. The peptide film was resuspended in DMSO to 5 mM and sonicated for 10 minutes, and then diluted in cold Ham’s F-12 (Sigma-Aldrich) to 100 μM, vortexed briefly, sonicated, and incubated at 4°C for 24 hours to generate oligomeric Aβ42.

### Immunocytochemistry.

After blocking, primary cultured hippocampal neurons were incubated with primary antibodies overnight at room temperature. After 4 washes with PBS, the neurons were incubated with Alexa Fluor–conjugated secondary antibodies (1:400 dilution of Alexa Fluor 488, 594, or 647 conjugates). Nuclei were labeled using DAPI. Images were acquired using a Nikon Ti2 confocal microscope and processed/analyzed with Nikon NLS element software. Synaptic density was determined by calculating the number of overlapping vGlut1/PSD95 (presynaptic and postsynaptic markers, respectively) puncta per unit length of dendrite reconstructed.

### Electrophysiology.

A whole-cell patch-clamp recording technique was used to measure synaptic transmission in pyramidal neurons from the hippocampal CA1 region. Briefly, animals were anesthetized and perfused with ice-cold oxygenated (95% O_2_ and 5% CO_2_) sucrose solution (220 mM sucrose, 15 mM HEPES, 11 mM glucose, 1 mM Na_2_HPO_4_, 4 mM MgSO_4_, 0.1 mM CaCl_2_, 2.5 mM KCl, pH 7.35, 300 mOsm). Brain tissues were rapidly removed, iced, and cut into 300 μm coronal slices using a vibratome (VT1000s, Leica). Brain slices were allowed to recover in oxygenated artificial cerebrospinal fluid (ACSF) (130 mM NaCl, 26 mM NaHCO_3_, 3 mM KCl, 5 mM MgCl_2_, 1.25 mM NaH_2_PO_4_, 1 mM CaCl_2_, 10 mM glucose, pH 7.60, 300 mOsm) for 40 minutes in a 33°C water bath and then kept at room temperature for 30 minutes prior to the start of recording. The slices were positioned in a perfusion chamber and submerged in continuously flowing oxygenated ACSF (2 mL/min). Pyramidal neurons were visualized using a 40× water-immersed lens with an upright microscope (Scientifica). Patch pipettes (3–6 MΩ) were pulled from 1.5 mm borosilicate glass capillaries using a micropipette puller (P-1000, Sutter Instrument) and filled with internal solution containing 130 mM Cs-methanesulfonate, 10 mM CsCl, 4 mM NaCl, 10 mM HEPES, 1 mM MgCl_2_, 5 mM EGTA, 2 mM QX-314, 12 mM phosphocreatine, 5 mM MgATP, and 0.2 mM Na_2_GTP (pH 7.2-7.3, 265-270 mOsm).

During recording, the brain slices were perfused with oxygenated ACSF (1 mL/min), and bicuculline (20 μM) was added to isolate excitatory transmission. Changes before and after 15 minutes of BafA1 (200 nM) bath application were recorded. sEPSCs were recorded by holding neurons at –70 mV. Presynaptic glutamate release was measured using PPR. Evoked EPSCs (eEPSCs) were elicited by local stimulation generated by a stimulation isolator controlled by a pulse generator (A-M Systems). Paired eEPSCs were measured across a range of interpulse intervals (20, 50, 100, 200, and 400 ms). Stimulation pulses (0.06 ms, 70 μA) were delivered at 0.05 Hz to minimize short-term synaptic plasticity, and 5 traces of paired eEPSCs were averaged. PPR was calculated by dividing the average amplitude of the second peak by the average amplitude of the first peak. To ensure the stimulus was similar across different slices, the tip of the bipolar electrode (FHC, Inc.) was carefully adjusted to approximately 20 μm below the surface of each slice (61) and 100 μm horizontally from the recorded cell (62, 63).

To minimize experimental variations, only cells with comparable membrane capacitances and series resistance changed by less than 10% were included. Data were acquired using a Multiclamp 700B amplifier (Molecular Devices) and digitized with a DigiData 1550B data acquisition board (Molecular Devices). Cells were allowed to stabilize for at least 2 minutes before recording. The recorded signals were digitized at 5 kHz, filtered at 1 kHz, and collected using a Clampex 11.0 data acquisition system (Molecular Devices). Data were analyzed with Clampfit 11.0 software (Molecular Devices).

### Transmission electron microscopy.

Copper grids (200 mesh with carbon-coated Formvar film) were activated in ethanol for 20 minutes at room temperature (RT). Concurrently, the SV samples were fixed with an equal volume of 4% formaldehyde for 10 minutes at RT. The samples were then incubated on the prepared grids for 1 hour at RT, followed by 3 washes with PBS, 10 minutes each. Negative staining was performed by incubating the grids twice (10 minutes each) with uranyl acetate replacement stain at RT. The grids were air-dried for 30 minutes at RT and imaged using an FEI Tecnai BioTwin transmission electron microscope at an accelerating voltage of 80 kV and a magnification of 32.4 kx.

### Statistics.

Data are displayed in box-and-whisker plots with all individual values shown. Results are expressed as minimum to maximum with median in the center. All statistical analysis was performed using GraphPad Prism 10 software. An unpaired, 2-tailed Student’s *t* test was used for comparisons between 2 groups when the samples were normally distributed and of equal variance. An unpaired 2-tailed *t* test with Welch’s correction was applied when both groups of data followed a normal distribution but were of unequal variance. A 2-tailed Mann-Whitney *U* test was performed when the samples were not normally distributed. One-way ANOVA, 2-way ANOVA, or multi-way ANOVA followed by Bonferroni’s post hoc analysis were used for multigroup comparisons as appropriate, unless otherwise stated. Correlation analyses were performed using Pearson’s correlation coefficient for continuous variables or Spearman’s rank correlation coefficient for categorical variables. The ROC curve was measured to determine the discriminating power of the proposed model. The AUC for each of the ROC curves is annotated with the 95% CI by the Wilson/Brown method. Fisher’s exact test was performed in binary categorical data. A *P* value of less than 0.05 was considered statistically significant, and *P* values are shown with original numbers or symbols as indicated in figures and figure legends.

### Study approval.

Animal studies were approved and performed following IACUC guidelines at the University of Kansas and Wake Forest University School of Medicine. Mice were kept in compliance with the NIH *Guide for the Care and Use of Laboratory Animals* (National Academies Press, 2011). Frozen postmortem temporal lobe tissues were obtained from the Brain and Body Donation Program at Banner Sun Health Research Institute (Sun City, Arizona, USA) and the University of Kansas Alzheimer’s Disease Research Center (KUADRC; Kansas City, Kansas, USA). Human tissue collection and distribution were conducted under institutionally approved protocols at the respective centers. Written informed consent was obtained from all participants. All procedures adhered to the Declaration of Helsinki.

### Data availability.

All data shown in the figures are available in the Supporting Data Values file.

## Author contributions

YC, KAFE, SY, JT, TW, and AP carried out experiments and collected the data. YC, KAFE, and TW collected samples and conducted the Western blot. ZJW and SY performed electrophysiology. YC, GD, and SM performed TEM. HD performed the V-ATPase activity. LG performed the acidification assay. YC, SY, LG, JT, and HD performed statistical analyses. LG and HD conceived the project, supervised the experiments, and wrote the manuscript. Review and editing of the manuscript was contributed by LG, HD, YC, and TW.

## Conflict of interest

The authors have declared that no conflict of interest exists.

## Funding support

This work is the result of NIH funding, in whole or in part, and is subject to the NIH Public Access Policy. Through acceptance of this federal funding, the NIH has been given a right to make the work publicly available in PubMed Central.

- NIH (R01AG059753 and R01AG075108 to HD).
- BrightFocus Foundation research grant (A20201159S to HD, A2022036S to LG).
- NIH grant P30 AG072973 to the University of Kansas Alzheimer’s Disease Research Center’s Research Education Component (REC fellowship to JT).
- Kansas INBRE (P20GM103418 to AP).

## Supplementary Material

Supplemental data

Supporting data values

## Figures and Tables

**Figure 1:**
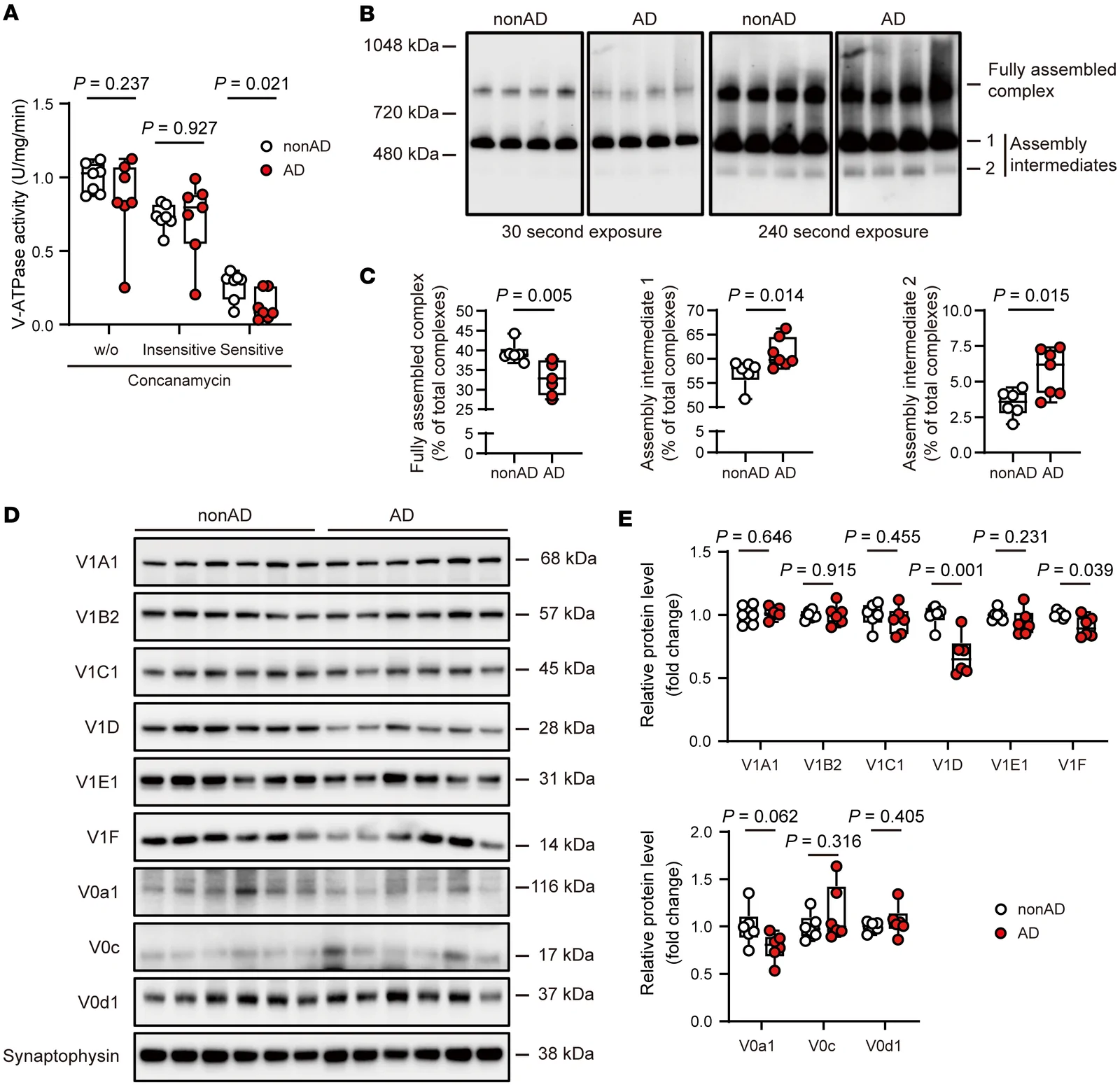
V-ATPase dysfunction in SV-rich fractions from patients with AD. (**A**) Measurement of V-ATPase ATP hydrolysis capacity in SV-rich fractions: 200 nM concanamycin was used as a selective V-ATPase inhibitor to determine the concanamycin-sensitive V-ATPase activity. Unpaired 2-tailed *t* test. *n* = 7 per group in controls without AD and patients with AD. (**B** and **C**) BN-PAGE and immunoblotting to examine V-ATPase fully assembled complexes and assembly intermediates based on detection of V0c subunit. (**B**) Representative immunoblot images. (**C**) Quantification of the proportion of the fully assembled complex and assembly intermediates. The fully assembled complex and intermediate 1 were analyzed using the Mann-Whitney *U* test; intermediate 2 was analyzed using an unpaired 2-tailed *t* test. Controls without AD: *n* = 6; patients with AD: *n* = 7. (**D** and **E**) Immunoblot analysis of key subunits of the V-ATPase complexes in SV-rich fractions. (**D**) Representative immunoblot images. (**E**) Quantification of the expression level of key V-ATPase subunits. Synaptophysin was used as a loading control. The expression levels of V-ATPase subunits in patients with AD were normalized to those in controls without AD. Unpaired 2-tailed *t* test, controls without AD, *n* = 6; patients with AD, *n* = 6.

**Figure 2:**
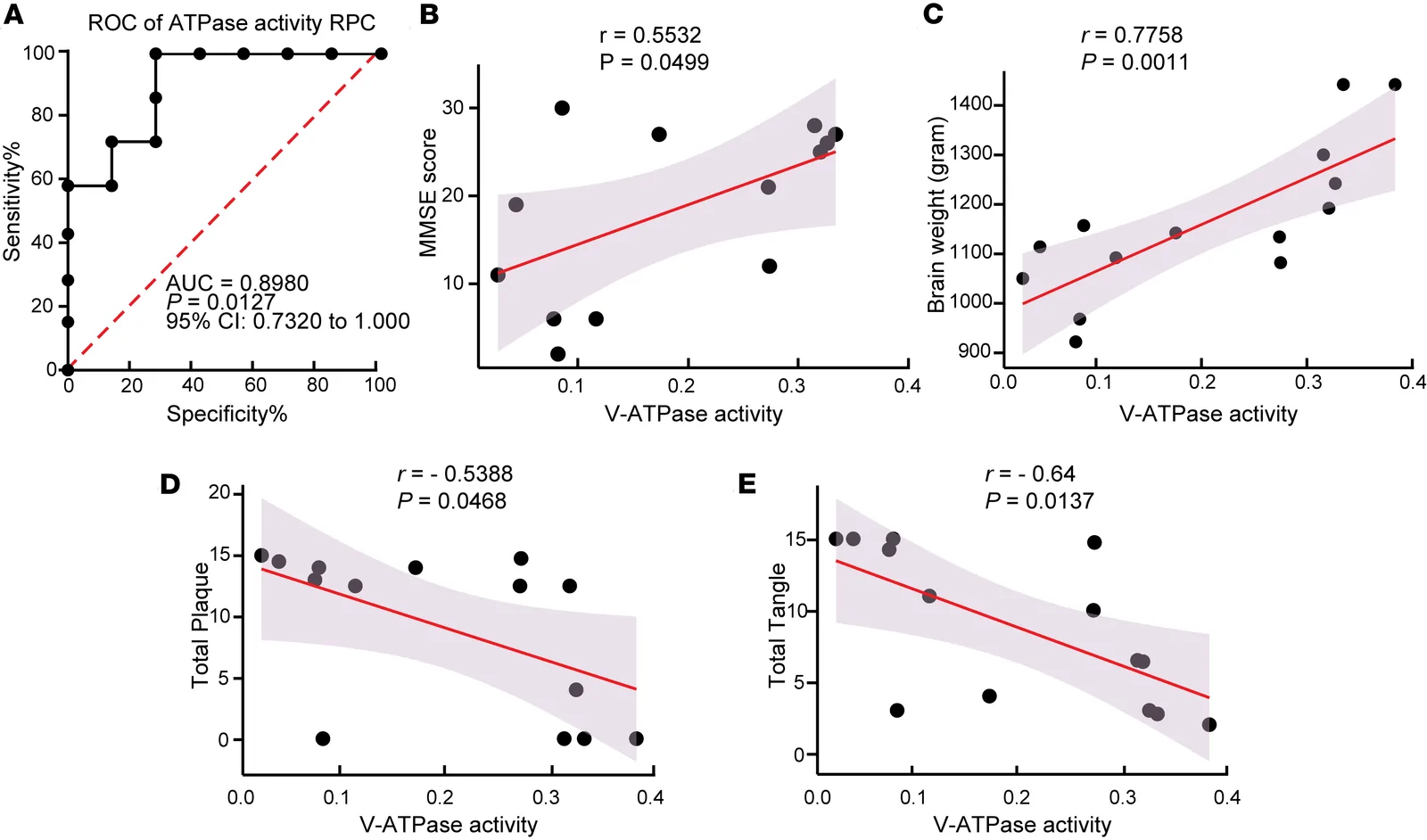
Clinicopathological relationship of V-ATPase dysfunction in SVs with cognitive deficits in AD. (**A**) ROC curves for SV-associated V-ATPase activity and AD diagnosis. (**B**–**E**) Correlation analysis between SV-associated V-ATPase activity and Mini-Mental State Examination (MMSE) score (**B**), brain weight (**C**), brain amyloidosis (**D**), and neurofibrillary tangle burden (**E**). Pearson’s correlation coefficients were used for correlation analysis. The shaded area (purple) represents mean ± 95% CI. *n* = 13–14.

**Figure 3:**
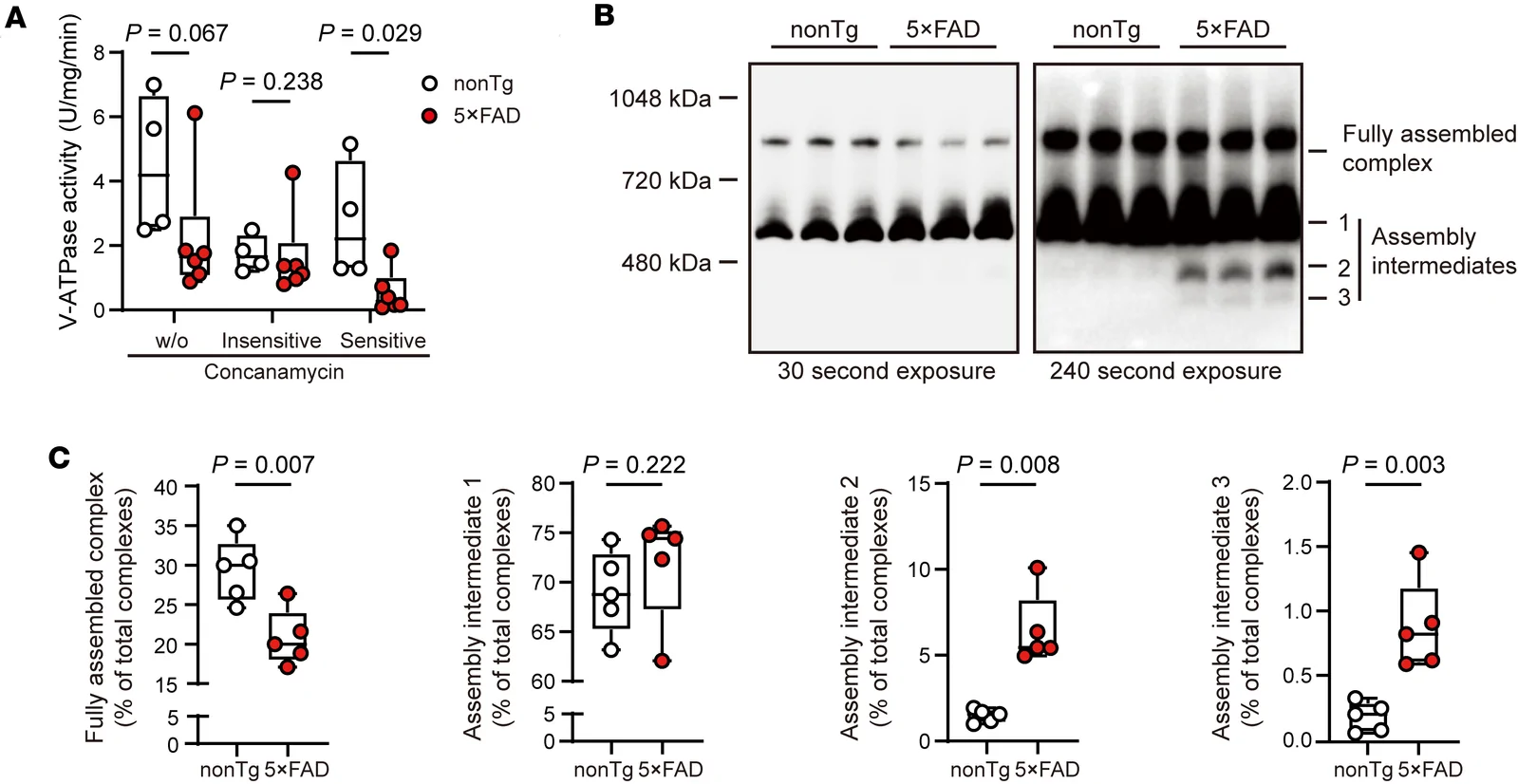
V-ATPase dysfunction in SV-rich fractions from 8-month-old 5×FAD mice. (**A**) Quantification of total, concanamycin-insensitive, and concanamycin-sensitive V-ATPase activities in SV-rich fractions. Mann-Whitney *U* test, Non-Tg, *n* = 4; 5×FAD, *n* = 6. (**B** and **C**) Assessment of V-ATPase complex assembly in SV-rich fractions based on detection of V0c subunit. (**B**) Representative immunoblot images. (**C**) Quantification of the proportions of the fully assembled complex and assembly intermediates. The fully assembled complex and intermediate 3 were analyzed using an unpaired 2-tailed *t* test; intermediate 1 and 2 were analyzed using Mann-Whitney *U* tests, *n* = 5 per group.

**Figure 4:**
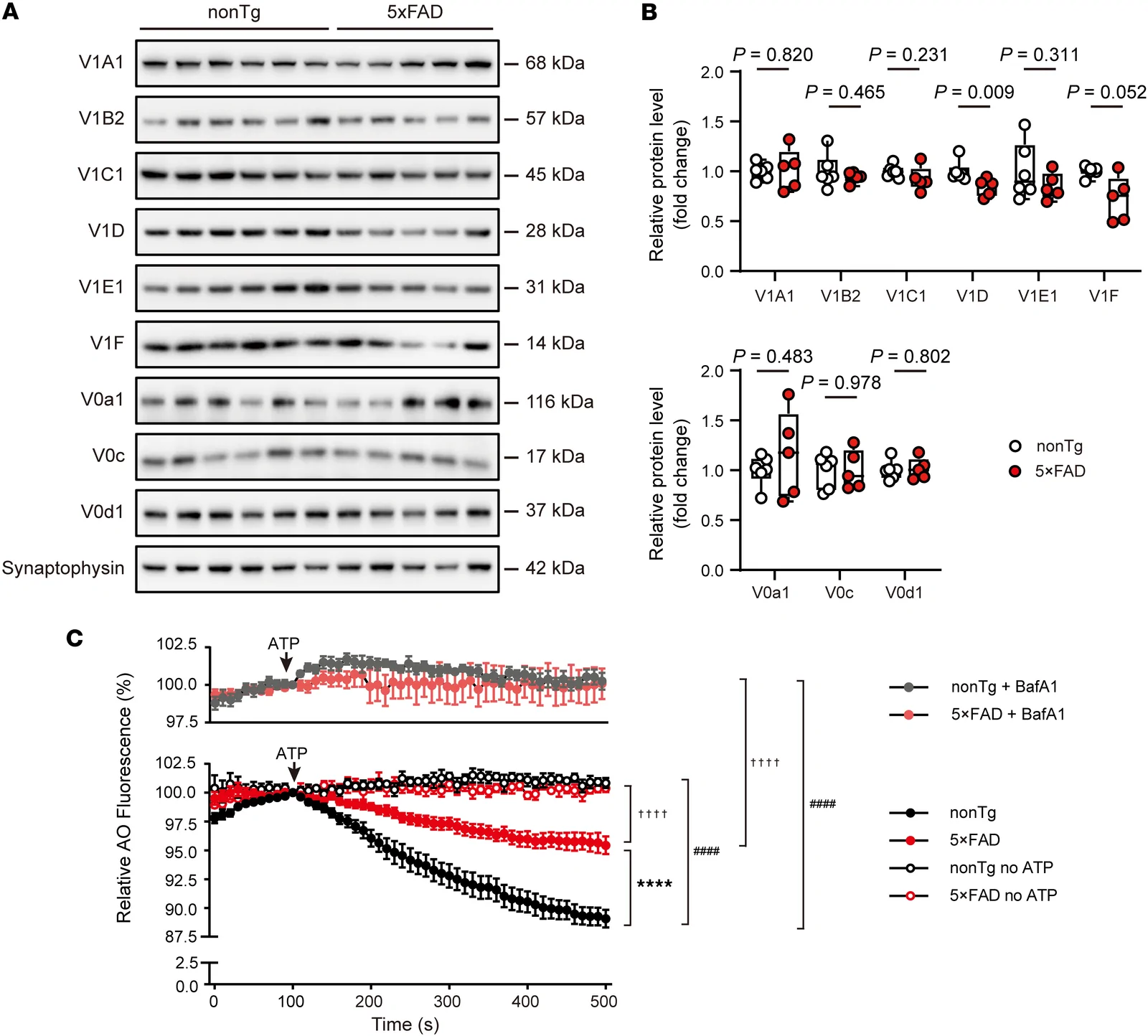
Reduced V1D expression and impaired acidification in SV-rich fractions from 8-month-old 5×FAD mice. (**A** and **B**) Immunoblot analysis of key subunits of the V-ATPase complex in SV-rich fractions from non-Tg and 5×FAD mice. (**A**) Representative Western blot images. (**B**) Quantification of the expression levels of key V-ATPase subunits in 5×FAD mice relative to non-Tg controls, with synaptophysin as a loading control. The V1D was analyzed using Mann-Whitney *U* test, and other subunits were analyzed using an unpaired 2-tailed *t* test. Non-Tg, *n* = 6; 5×FAD, *n* = 5. (**C**) SV acidification assessed by acridine orange (AO) fluorescence SV-rich fractions over a 500-second time course. Data are presented as mean ± SEM. Two-way ANOVA followed by Bonferroni’s post hoc test. Non-Tg versus non-Tg no ATP; non-Tg versus non-Tg + BafA1, ^####^*P* < 0.0001. 5×FAD versus 5×FAD no ATP; 5×FAD versus 5×FAD + BafA1, ^††††^*P* < 0.0001. Non-Tg versus 5×FAD, ****P < 0.0001.

**Figure 5:**
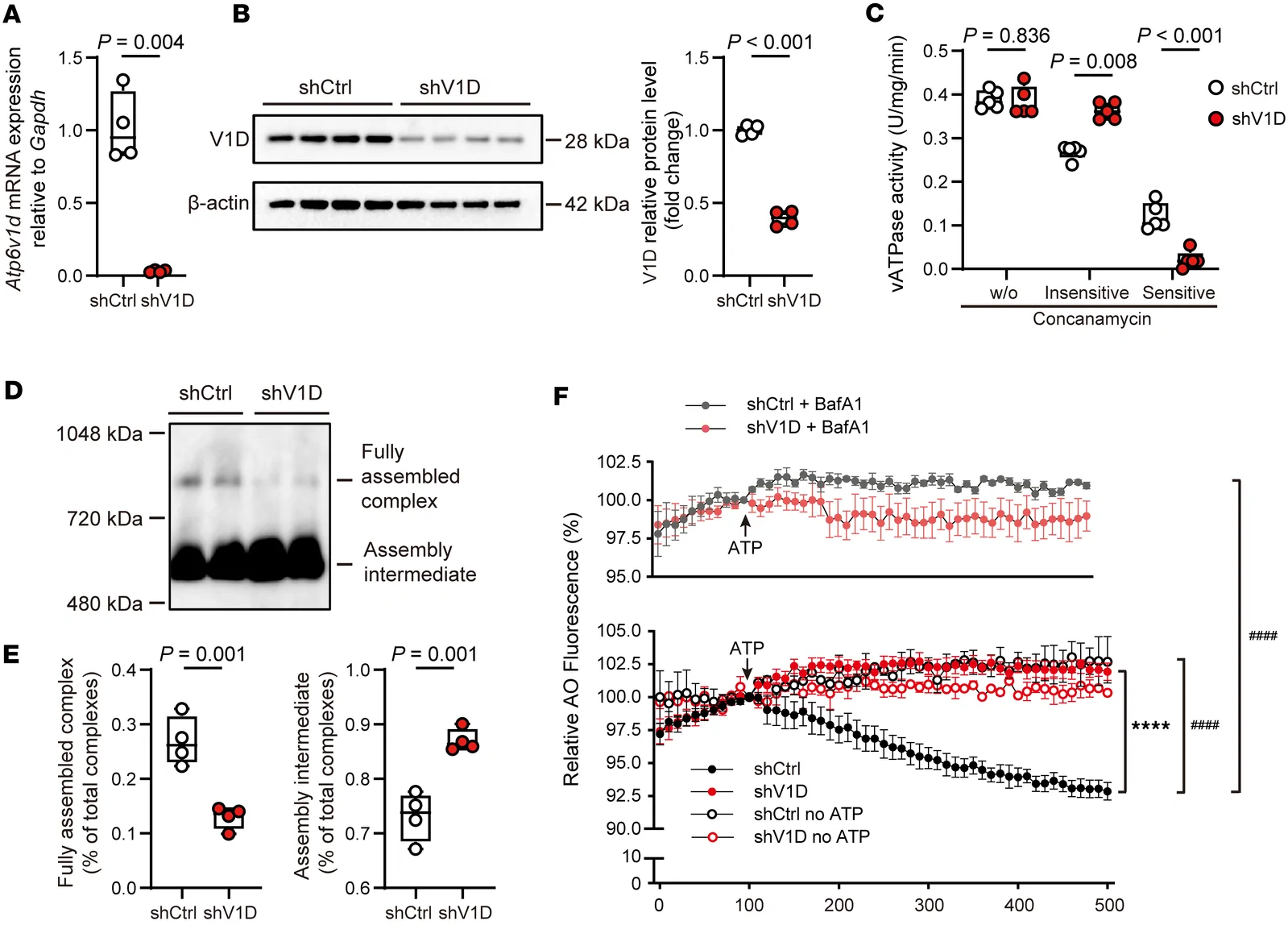
Impaired SV proton transport in V1D-deficient primary cultured neurons. (**A** and **B**) V1D expression in primary cultured neurons after infection with lentivirus carrying *Atp6v1d*-targeted shRNA (shV1D) or nontargeted scramble control shRNA (shCtrl), assessed at both mRNA (**A**) and protein (**B**) levels. Quantification of (**A**) *Atp6v1d* mRNA normalized to *Gapdh* and (**B**) V1D protein levels normalized to β-actin. Unpaired 2-tailed *t* test, *n* = 4 per group. (**C**) Reduced concanamycin-sensitive V-ATPase activity in *Atp6v1d* shRNA-treated neurons relative to scramble control shRNA. Statistical significance was assessed using either unpaired 2-tailed *t* test or Mann-Whitney *U* test, as appropriate. *n* = 5 per group. (**D** and **E**) Examination of V-ATPase complex assembly in primary cultured neurons. Unpaired 2-tailed *t* test, *n* = 4 per group. (**F**) SV acidification assessed by AO fluorescence SV-rich fractions over a 500-second time course. Data are presented as mean ± SEM. Two-way ANOVA followed by Bonferroni’s post hoc test. shCtrl versus shCtrl no ATP; shCtrl versus shCtrl + BafA1, ^####^*P* < 0.0001. shCtrl versus shV1D, ****P < 0.0001.

**Figure 6:**
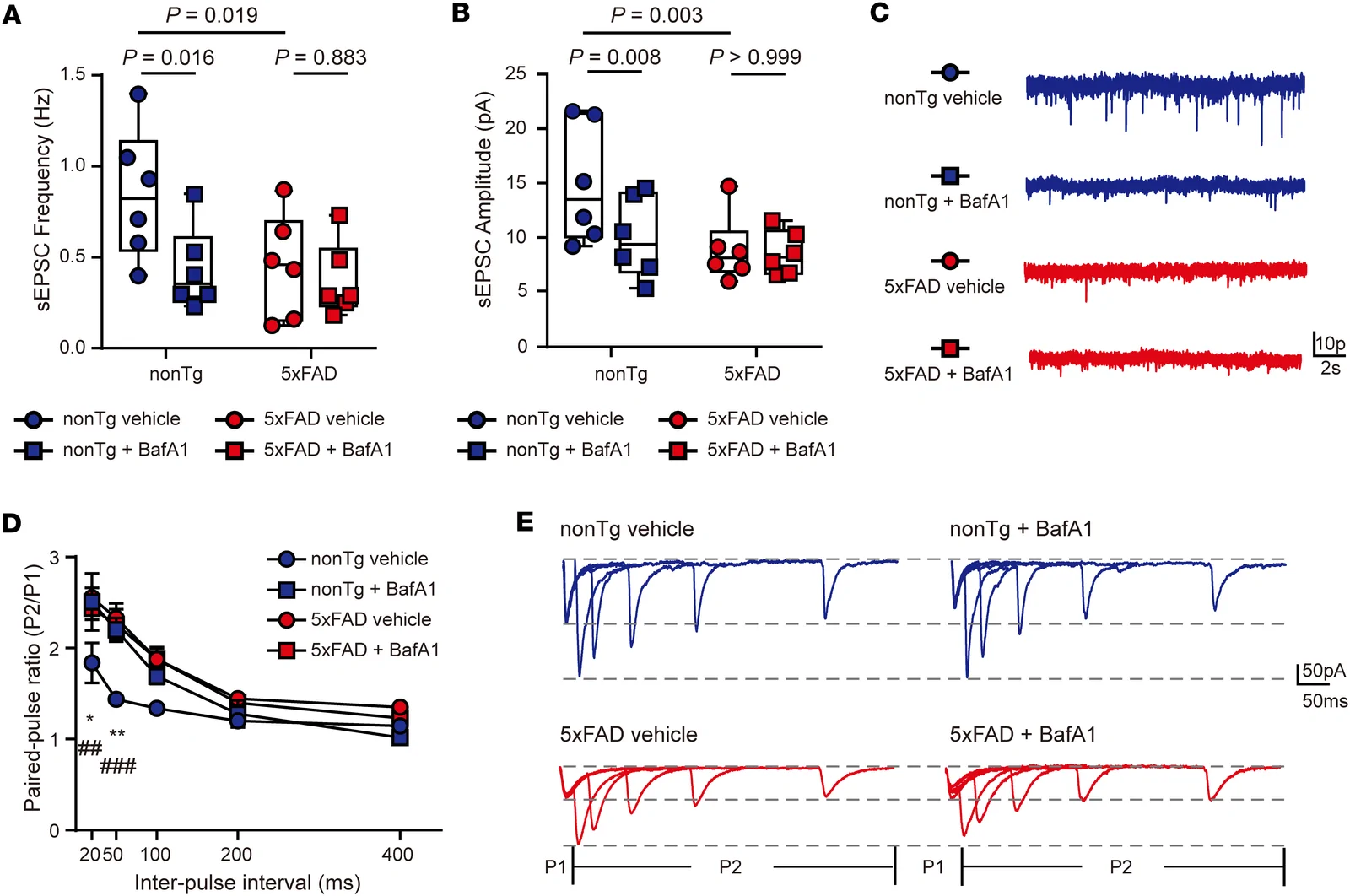
Impact of disrupted V-ATPase proton transport on synaptic transmission in 8-month-old 5×FAD mice. (**A** and **B**) Averaged sEPSC frequency (**A**) and amplitude (**B**) in pyramidal neurons from 8-month-old non-Tg and 5×FAD mice before and after treatment with vehicle or 200 nM BafA1. Two-way ANOVA followed by Bonferroni’s post hoc test. Non-Tg: *n* = 6 cells from 3 mice; 5×FAD: *n* = 6 cells from 3 mice. (**C**) Representative sEPSC traces before and after treatment. (**D**) Measurement of the PPR in pyramidal neurons from both non-Tg and 5×FAD mice. Recordings were conducted before and after applying vehicle or 200 nM BafA1 at various interstimulus intervals of 20, 50, 100, 200, and 400 ms. Multi-way ANOVA followed by Bonferroni’s post hoc test. Non-Tg: *n* = 8 cells from 4 mice, 5×FAD: *n* = 7 cells from 4 mice. Non-Tg vehicle versus non-Tg BafA1: *P < 0.05, **P < 0.01. Non-Tg vehicle versus 5×FAD vehicle: ^##^*P* < 0.01, ^###^*P* < 0.001. (**E**) Representative PPR traces before and after 200 nM BafA1.

**Figure 7:**
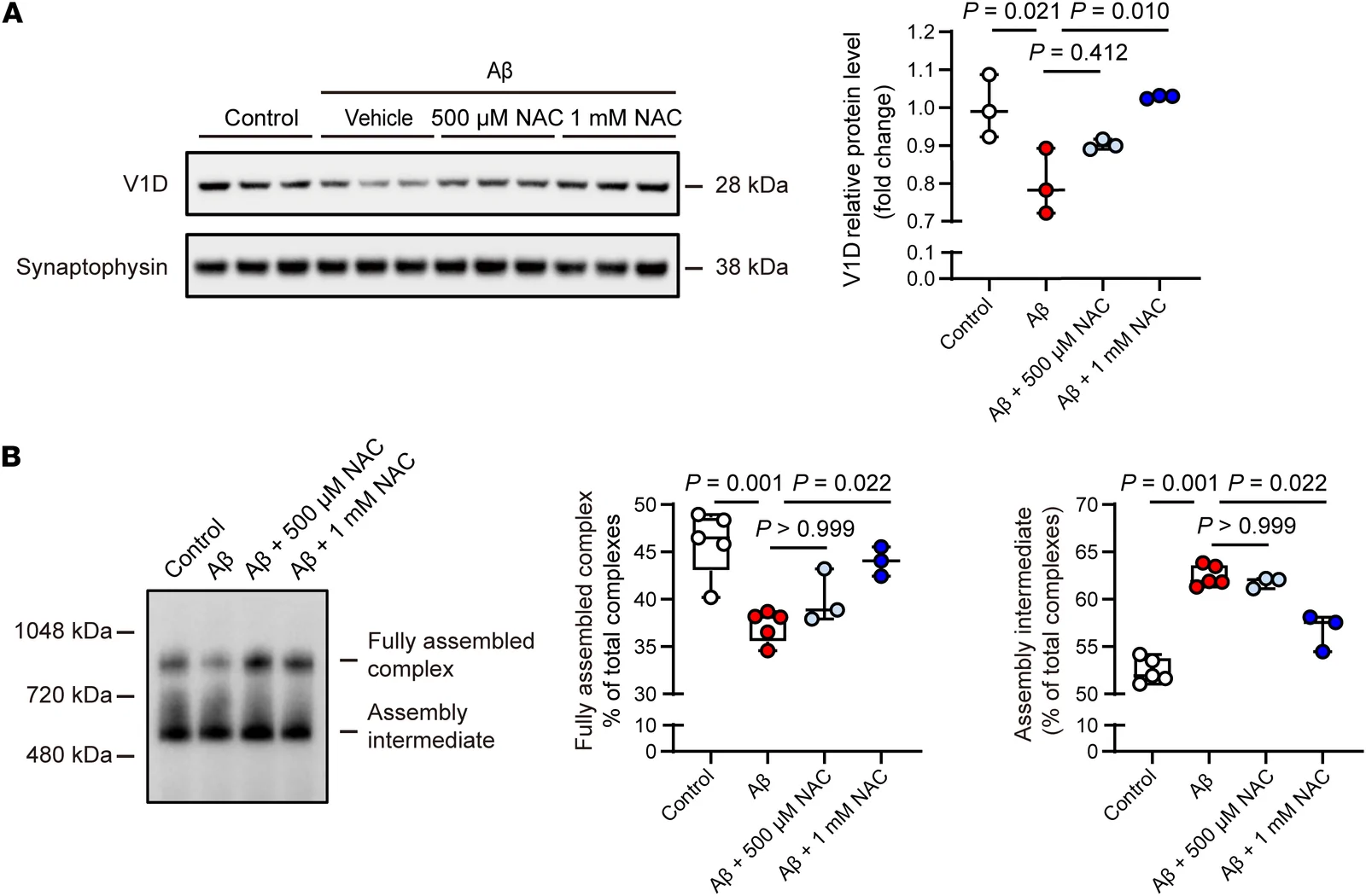
Aβ-induced oxidative stress promotes V-ATPase disassembly in SVs via V1D loss. (**A**) Immunoblot analysis of V1D in SV-rich fractions from ex vivo–cultured organotypic brain slices treated with oligomeric Aβ42 (500 μM) and/or NAC (500 μM or 1 mM), normalized to synaptophysin. One-way ANOVA followed by Bonferroni’s post hoc test. *n* = 3 per group. (**B**) Assessment of V-ATPase complex assembly in SV-rich fractions based on detection of V0c subunit. One-way ANOVA followed by Bonferroni’s post hoc test. *n* = 5 in control and Aβ groups; *n* = 3 in Aβ + NAC 500 μM and Aβ + NAC 1 mM groups.

## Version 1 10/08/2026

Electronic publication

## References

[1] Selkoe DJ (2002). Alzheimer’s disease is a synaptic failure. Science.

[2] Martinez-Serra R (2022). Emerging insights into synapse dysregulation in Alzheimer’s disease. Brain Commun.

[3] Meftah S, Gan J (2023). Alzheimer’s disease as a synaptopathy: evidence for dysfunction of synapses during disease progression. Front Synaptic Neurosci.

[4] Ferreira-Vieira TH (2016). Alzheimer’s disease: targeting the cholinergic system. Curr Neuropharmacol.

[5] Conway ME (2020). Alzheimer’s disease: targeting the glutamatergic system. Biogerontology.

[6] Tyler WJ, Murthy VN (2004). Synaptic vesicles. Curr Biol.

[7] Lan G (2022). Association of presynaptic loss with Alzheimer’s disease and cognitive decline. Ann Neurol.

[8] Anschuetz A (2025). Proteomic and non-proteomic changes of presynaptic proteins in animal models of Alzheimer’s disease: a meta-analysis 2015-2023. J Alzheimers Dis.

[9] Parodi J (2010). Beta-amyloid causes depletion of synaptic vesicles leading to neurotransmission failure. J Biol Chem.

[10] Chen MK (2018). Assessing synaptic density in Alzheimer disease with synaptic vesicle glycoprotein 2A positron emission tomographic imaging. JAMA Neurol.

[11] Cross RL, Muller V (2004). The evolution of A-, F-, and V-type ATP synthases and ATPases: reversals in function and changes in the H+/ATP coupling ratio. FEBS Lett.

[12] McGuire C (2016). Regulation of V-ATPase assembly and function of V-ATPases in tumor cell invasiveness. Biochim Biophys Acta.

[13] Breton S, Brown D (2013). Regulation of luminal acidification by the V-ATPase. Physiology (Bethesda).

[14] Zhao J (2015). Electron cryomicroscopy observation of rotational states in a eukaryotic V-ATPase. Nature.

[15] Mazhab-Jafari MT (2016). Atomic model for the membrane-embedded V_O_ motor of a eukaryotic V-ATPase. Nature.

[16] Mindell JA (2012). Lysosomal acidification mechanisms. Annu Rev Physiol.

[17] Wang C (2024). Structure and topography of the synaptic V-ATPase-synaptophysin complex. Nature.

[18] Coupland CE (2024). High-resolution electron cryomicroscopy of V-ATPase in native synaptic vesicles. Science.

[19] Wang B (2024). TFEB-vacuolar ATPase signaling regulates lysosomal function and microglial activation in tauopathy. Nat Neurosci.

[20] Im E (2023). Lysosomal dysfunction in Down syndrome and Alzheimer mouse models is caused by v-ATPase inhibition by Tyr^682^-phosphorylated APP βCTF. Sci Adv.

[21] Lee JH (2022). Faulty autolysosome acidification in Alzheimer’s disease mouse models induces autophagic build-up of Aβ in neurons, yielding senile plaques. Nat Neurosci.

[22] Torres M (2012). Defective lysosomal proteolysis and axonal transport are early pathogenic events that worsen with age leading to increased APP metabolism and synaptic Abeta in transgenic APP/PS1 hippocampus. Mol Neurodegener.

[23] Kim SH (2023). Endolysosomal impairment by binding of amyloid beta or MAPT/Tau to V-ATPase and rescue via the HYAL-CD44 axis in Alzheimer disease. Autophagy.

[24] Wang M (2021). Transformative network modeling of multi-omics data reveals detailed circuits, key regulators, and potential therapeutics for Alzheimer’s disease. Neuron.

[25] de Flores R (2022). Medial temporal lobe networks in Alzheimer’s disease: structural and molecular vulnerabilities. J Neurosci.

[26] Jia K (2023). Mitochondria-sequestered Aβ renders synaptic mitochondria vulnerable in the elderly with a risk of Alzheimer disease. JCI Insight.

[27] Symersky J (2012). Oligomycin frames a common drug-binding site in the ATP synthase. Proc Natl Acad Sci U S A.

[28] Sanchez-Rodriguez JE (2015). A structural rearrangement of the Na+/K+-ATPase traps ouabain within the external ion permeation pathway. J Mol Biol.

[29] Sehgal P (2017). Inhibition of the sarco/endoplasmic reticulum (ER) Ca^2+^-ATPase by thapsigargin analogs induces cell death via ER Ca^2+^ depletion and the unfolded protein response. J Biol Chem.

[30] Huss M (2002). Concanamycin A, the specific inhibitor of V-ATPases, binds to the V(o) subunit c. J Biol Chem.

[31] Forgac M (2007). Vacuolar ATPases: rotary proton pumps in physiology and pathophysiology. Nat Rev Mol Cell Biol.

[32] Collins MP, Forgac M (2020). Regulation and function of V-ATPases in physiology and disease. Biochim Biophys Acta Biomembr.

[33] Eimer WA, Vassar R (2013). Neuron loss in the 5XFAD mouse model of Alzheimer’s disease correlates with intraneuronal Aβ42 accumulation and Caspase-3 activation. Mol Neurodegener.

[34] Tian J (2019). Disrupted hippocampal growth hormone secretagogue receptor 1α interaction with dopamine receptor D1 plays a role in Alzheimer’s disease. Sci Transl Med.

[35] Palmgren MG (1990). An H-ATPase assay: proton pumping and ATPase activity determined simultaneously in the same sample. Plant Physiol.

[36] Takamori S (2006). Molecular anatomy of a trafficking organelle. Cell.

[37] Turk V (2001). Lysosomal cysteine proteases: facts and opportunities. EMBO J.

[38] Wang R (2021). Molecular basis of V-ATPase inhibition by bafilomycin A1. Nat Commun.

[39] Stobrawa SM (2001). Disruption of ClC-3, a chloride channel expressed on synaptic vesicles, leads to a loss of the hippocampus. Neuron.

[40] Abe M (2019). Functional complementation reveals that 9 of the 13 human V-ATPase subunits can functionally substitute for their yeast orthologs. J Biol Chem.

[41] Bonnycastle K (2021). Presynaptic dysfunction in neurodevelopmental disorders: insights from the synaptic vesicle life cycle. J Neurochem.

[42] Gauba E (2021). Modulation of OSCP mitigates mitochondrial and synaptic deficits in a mouse model of Alzheimer’s pathology. Neurobiol Aging.

[43] Khan MM (2022). Oxidative stress protein Oxr1 promotes V-ATPase holoenzyme disassembly in catalytic activity-independent manner. EMBO J.

[44] Butterfield DA, Halliwell B (2019). Oxidative stress, dysfunctional glucose metabolism and Alzheimer disease. Nat Rev Neurosci.

[45] Jurcau A, Simion A (2020). Oxidative Stress in the pathogenesis of Alzheimer’s disease and cerebrovascular disease with therapeutic implications. CNS Neurol Disord Drug Targets.

[46] Samuni Y (2013). The chemistry and biological activities of N-acetylcysteine. Biochim Biophys Acta.

[47] Kennedy MB (2013). Synaptic signaling in learning and memory. Cold Spring Harb Perspect Biol.

[48] Wilson DM (2023). Hallmarks of neurodegenerative diseases. Cell.

[49] Wang B, Dudko OK (2021). A theory of synaptic transmission. Elife.

[50] Kodis EJ (2018). N-methyl-D-aspartate receptor-mediated calcium influx connects amyloid-β oligomers to ectopic neuronal cell cycle reentry in Alzheimer’s disease. Alzheimers Dement.

[51] O’Connor M (2020). Acetylation of AMPA receptors regulates receptor trafficking and rescues memory deficits in Alzheimer’s disease. iScience.

[52] Bodzeta A (2017). The presynaptic v-ATPase reversibly disassembles and thereby modulates exocytosis but is not part of the fusion machinery. Cell Rep.

[53] Haass C (2012). Trafficking and proteolytic processing of APP. Cold Spring Harb Perspect Med.

[54] Nixon RA (2017). Amyloid precursor protein and endosomal-lysosomal dysfunction in Alzheimer’s disease: inseparable partners in a multifactorial disease. FASEB J.

[55] Zhou Z (2021). Downregulation of ATP6V1A involved in Alzheimer’s disease via synaptic vesicle cycle, phagosome, and oxidative phosphorylation. Oxid Med Cell Longev.

[56] Fang F (2024). Neuronal hyperactivity in the hippocampus during the early stage of Streptozotocin-induced type 1 diabetes in mice. Neuroendocrinology.

[57] Wang Q (2019). Amyloid beta-mediated KIF5A deficiency disrupts anterograde axonal mitochondrial movement. Neurobiol Dis.

[58] Beck SJ (2016). Deregulation of mitochondrial F1FO-ATP synthase via OSCP in Alzheimer’s disease. Nat Commun.

[59] Massaro Tieze S (2022). Subcellular fractionation for the isolation of synaptic components from the murine brain. J Vis Exp.

[60] Kroener S (2009). Dopamine modulates persistent synaptic activity and enhances the signal-to-noise ratio in the prefrontal cortex. PLoS One.

[61] Liu Z (2017). IGF1-dependent synaptic plasticity of mitral cells in olfactory memory during social learning. Neuron.

[62] Wang ZJ (2020). Amelioration of autism-like social deficits by targeting histone methyltransferases EHMT1/2 in Shank3-deficient mice. Mol Psychiatry.

[63] Wang ZJ (2021). Autism risk gene KMT5B deficiency in prefrontal cortex induces synaptic dysfunction and social deficits via alterations of DNA repair and gene transcription. Neuropsychopharmacology.

