# Supplementary material for: Disrupted synaptic vacuolar H+-ATPase renders synaptic vulnerability to Alzheimer’s disease
Source: JCI Insight. 2026 Oct 8;11(19):e202983. doi: 10.1172/jci.insight.202983 (PMC13726408; doi:10.1172/jci.insight.202983)
Supplement: Supplementary file 1 [file jciinsight-11-202983-s230.pdf]

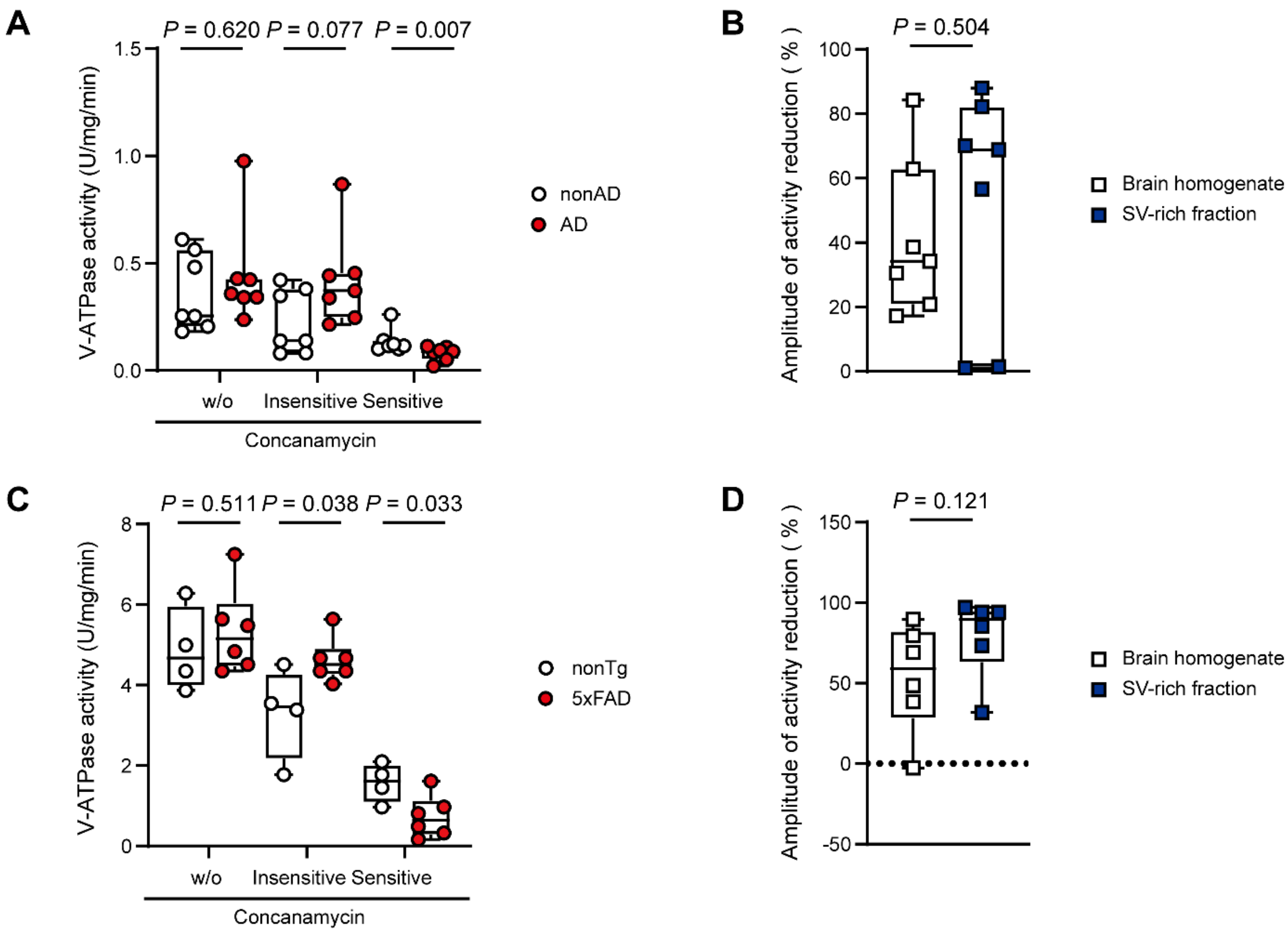

Supplemental Figure 1. Reduced V-ATPase activity in whole brain homogenates from patients with AD and 8-month-old 5×FAD mice. (A) Measurement of V-ATPase ATP hydrolysis capacity in brain homogenates from nonAD controls and AD patients. 200 nM Concanamycin was used as a selective V-ATPase inhibitor to determine the concanamycin-sensitive V-ATPase activity. n = 7 per group in both nonAD controls and AD patients. **(B)** Amplitude of V-ATPase activity reduction in AD patients versus non-AD controls **(C)** V-ATPase activity in brain homogenates from nonTg and 5×FAD mice. nonTg n = 4, 5×FAD n = 6. **(D)** Amplitude of V-ATPase activity reduction in 5×FAD versus nonTg controls. Statistical significance was determined by Unpaired two-tailed t test or Mann-Whitney U test, as appropriate.

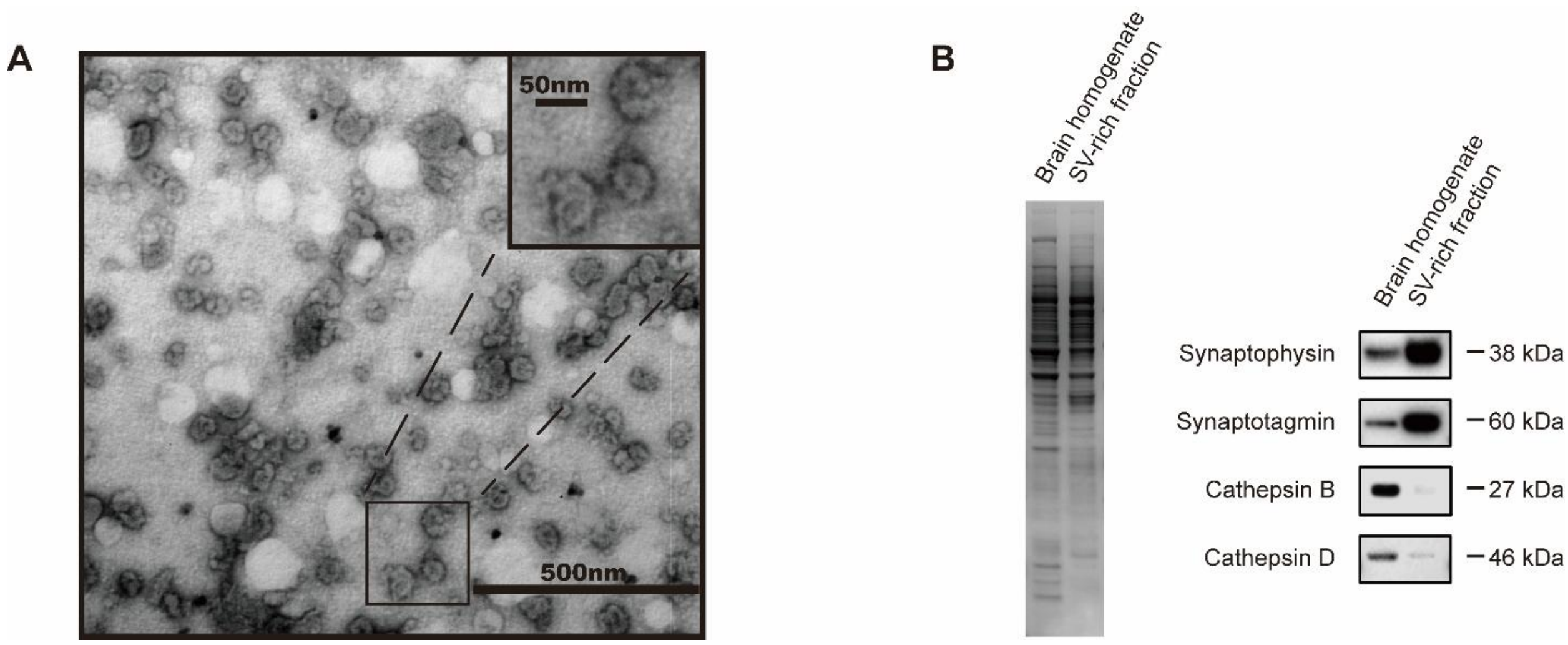

Supplemental Figure 2: SV-rich fraction Validation. (**A**) Transmission electron microscopy (TEM) image of SVs. scale bar = 500 nm (inset scale bar = 50 nm). (**B**) SV-rich fraction validation by SV markers. Left panel, Coomassie blue image of brain homogenate and SV-rich fraction; right panel, immunoblot images.

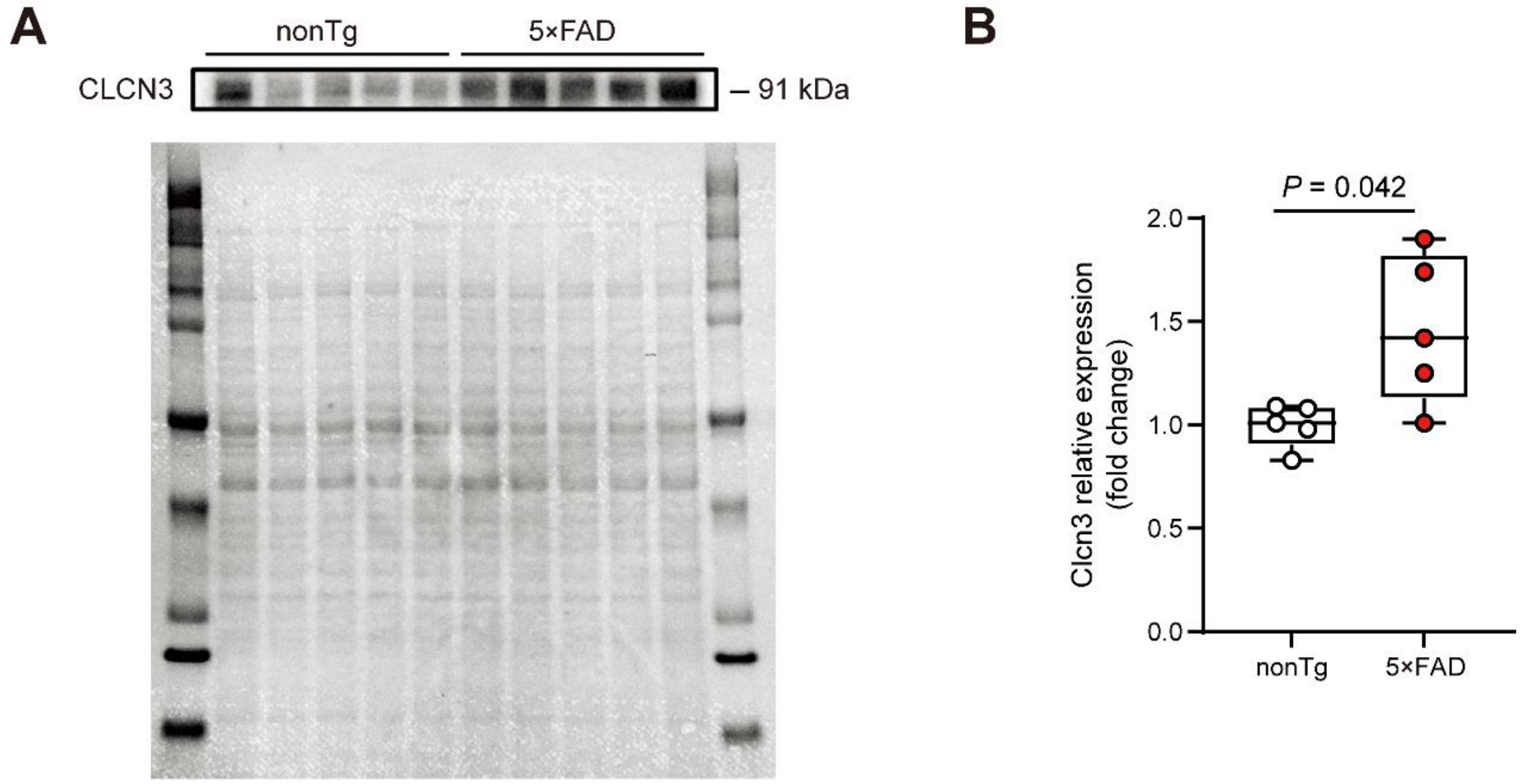

Supplemental Figure 3. Expression of chloride channel 3 (CLCN3) in nonTg and 5×FAD mouse SV- rich fractions. (**A**) Immunoblotting images of CLCN3 (upper image) and Ponceau S stain (lower image) in 8-month-old nonTg and 5×FAD mice SV-rich fractions. (**B**) CLCN3 expression determined by immunoblotting. Unpaired two-tailed *t* test. *n* = 5 per group.

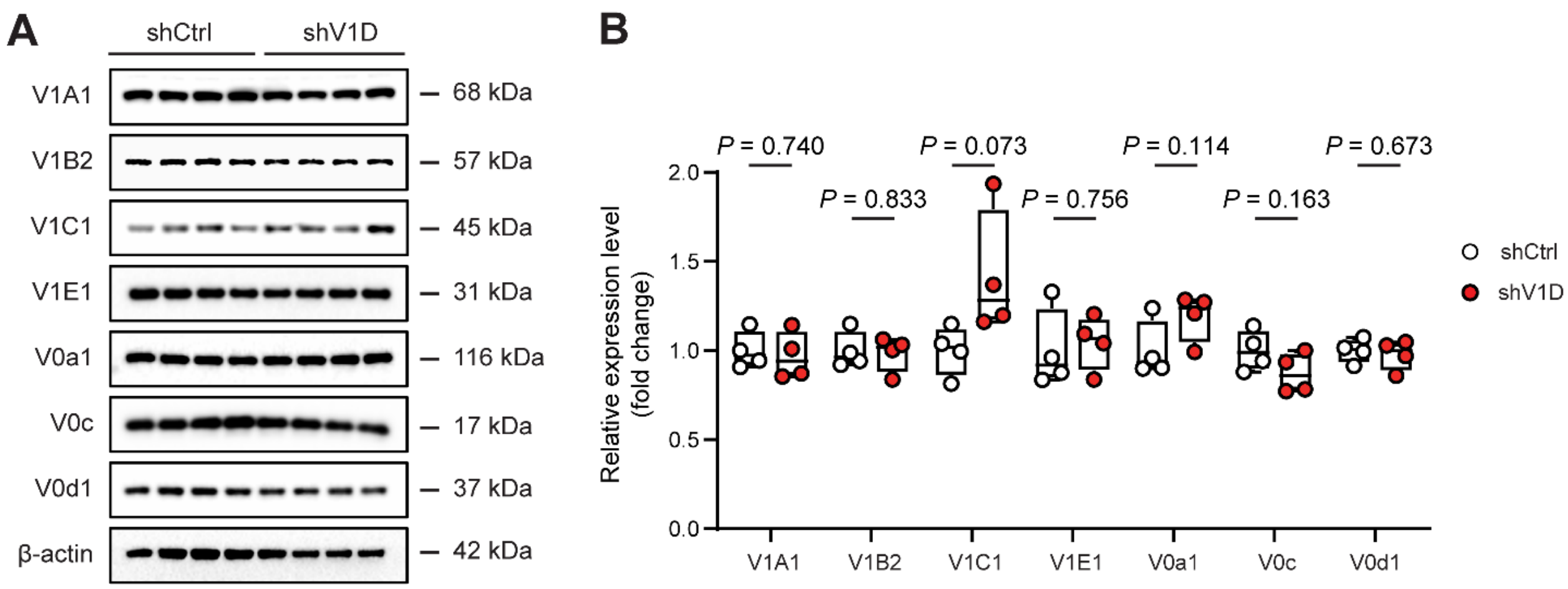

Supplemental Figure 4. Expression of V-ATPase complex subunits in primary cultured hippocampal neurons. (**A**) Immunoblot images and (**B**) quantification of V-ATPase subunit expression levels in *Atp6v1d* shRNA-treated primary hippocampal neurons (shV1D) relative to vehicle controls (shCtrl), with β-actin as a loading control. Statistical significance was determined by Unpaired two-tailed *t* test or Mann-Whitney U test, as appropriate. *n* = 4 per group.

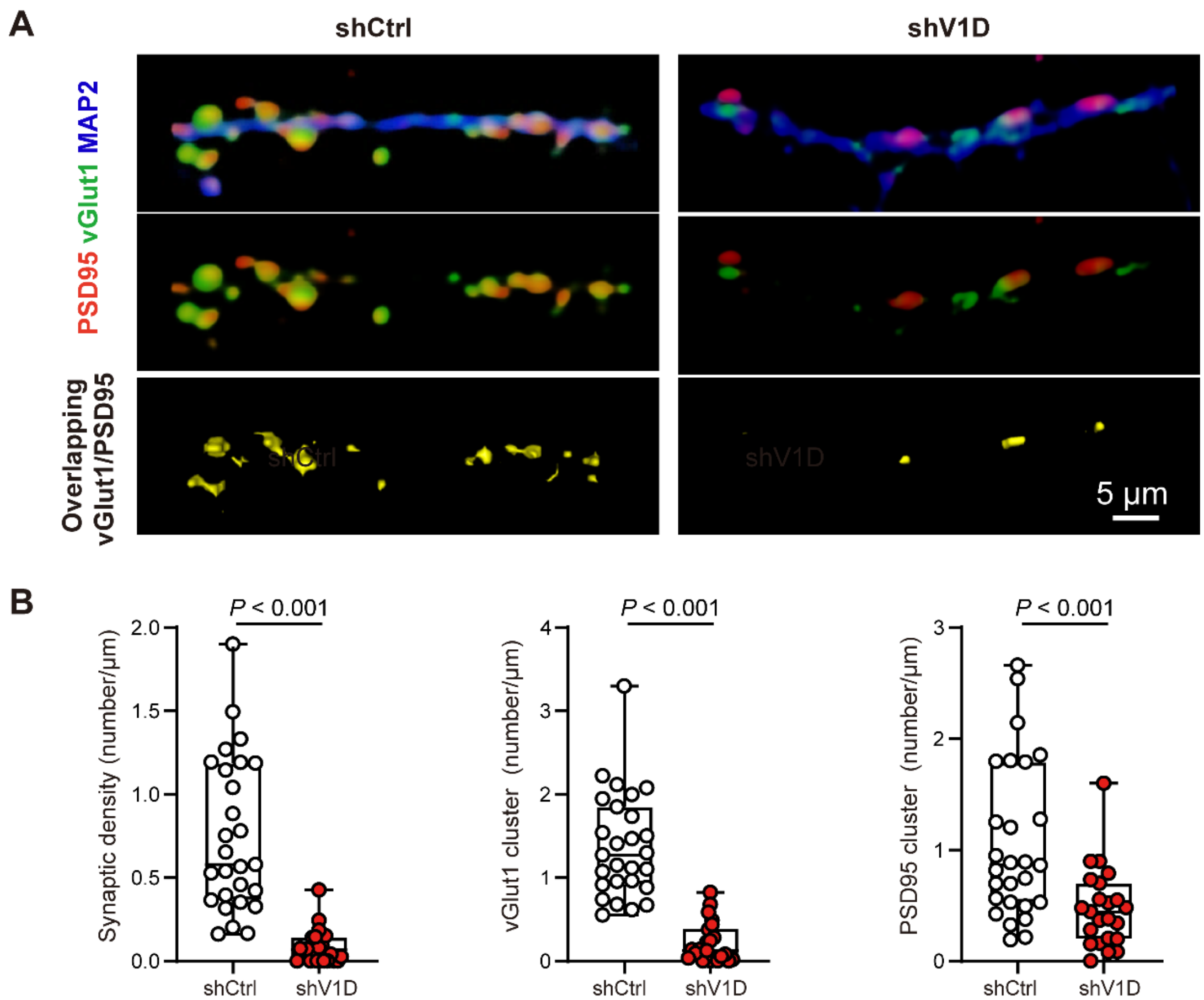

Supplemental Figure 5. Reduced synaptic density in primary cultured hippocampal neurons. (**A**) Representative immunofluorescence images of microtubule-associated protein 2 (MAP2, blue), postsynaptic density 95 (PSD95, red), and vesicular glutamate transporter 1 (vGlut1, green) in primary hippocampal neuron treated with either *Atp6v1d*-targeted shRNA (shV1D) or non-targeted scramble shRNA (shCtrl). scale bar = 5µm. (**B**) Quantitative analysis of synaptic density was determined by the number of overlapping vGlut1/PSD95 puncta per unit length of dendrite reconstructed. Mann–Whitney U test. shCtrl $n = 27$, shV1D $n = 22$.

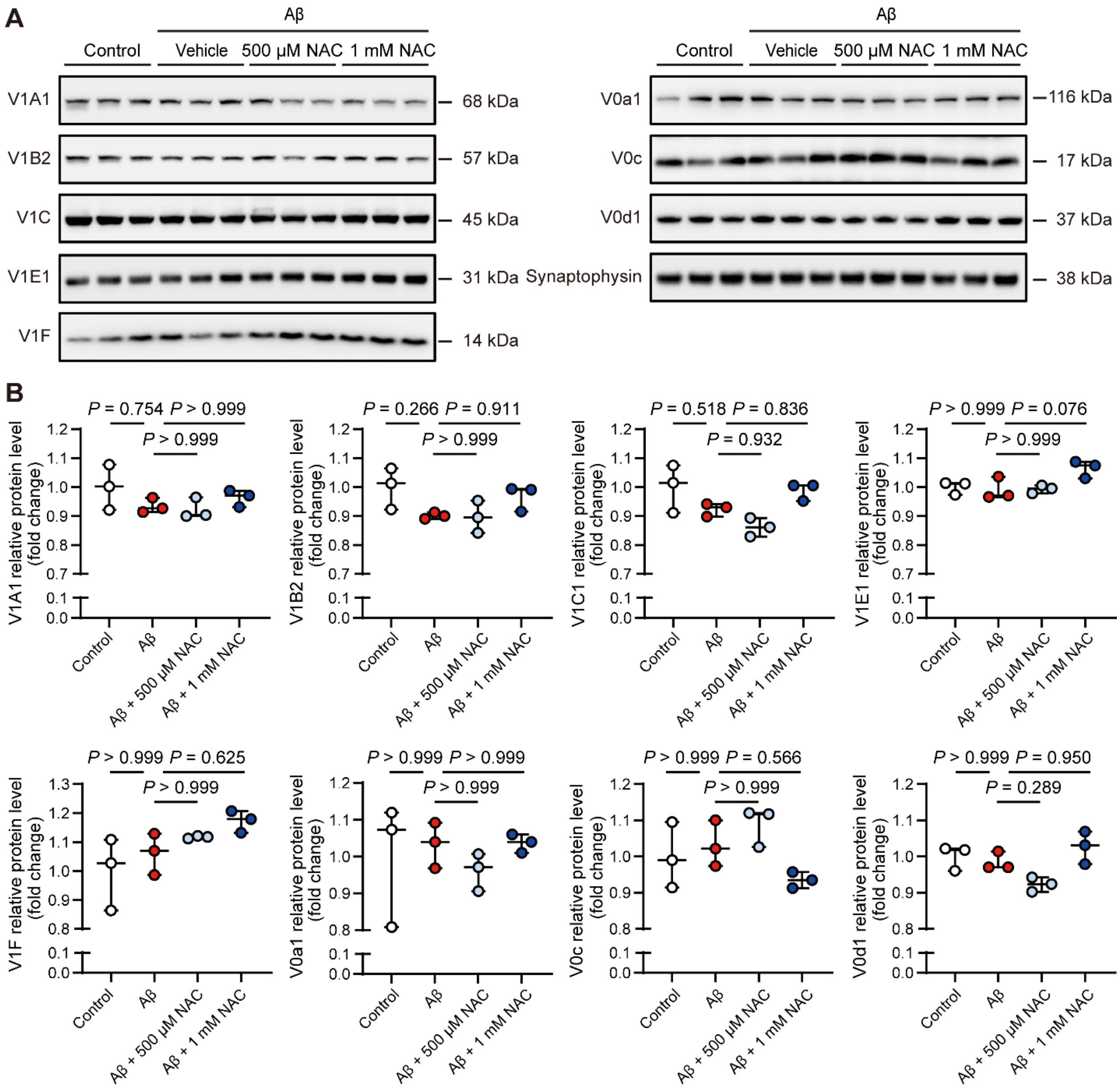

Supplemental Figure 6. Expression of V-ATPase complex subunits in SV-rich fractions from ex vivo cultured organotypic brain slices treated with Aβ and/or NAC. **(A)** Immunoblot images and (**B**) quantification of V-ATPase subunit expression levels in SV-rich fractions from ex vivo cultured organotypic brain slices treated with Aβ (500 µM) and/or NAC (500 µM or 1 mM), with synaptophysin as a loading control. Statistical significance was determined by One-way ANOVA followed by Bonferroni's post hoc or Kruskal-Wallis test followed by Dunn's multiple comparisons test as appropriate. *n* = 3 per group.

Supplemental Table 1. Demographic characteristics of patients.

Clinical Diagnosis	Case number	Gender[A]	Age[B]	Brain Weight[B] (gram)	Plaque total [C]	Thal stage[C]	Tangle total[C]	Braak	CERAD score
nonAD	05-16	M	82	1240	4	NA	3	III	0
nonAD	15-60	M	82	1155	0	NA	3	II	NA
nonAD	01-31	M	81	1190	12.5	NA	6.41	III	0
nonAD	12-44	F	80	1140	14	NA	4	III	2
nonAD	10-26	F	95	1298	0	NA	6.5	III	0
nonAD	05-10	M	91	1440	0	NA	2.75	II	0
nonAD	10-70	M	74	1440	0	NA	2	I	0
nonAD	00-03	M	82	1620	NA	2	NA	I	0
nonAD	00-05	M	89	1430	NA	0	NA	II	0
nonAD	00-14	M	91	NA	NA	3	NA	I	1
nonAD	00-40	M	74	NA	NA	4	NA	I	0
nonAD	00-44	M	67	NA	NA	1	NA	I	0
nonAD	00-54	M	95	NA	NA	0	NA	I	0
Mean ± 95% CI		2F/11M	83.31 ± 5.19	1328 ± 126	4.357 ± 5.793	1.167 ± 0.428	3.951 ± 1.672		0.25 ± 0.3949
AD	00-37	M	81	1090	12.5	NA	11	V	3
AD	11-27	M	89	1132	12.5	NA	10	V	3
AD	11-78	M	82	1112	14.5	NA	15	V	3
AD	03-06	F	89	920	13	NA	14.25	V	3
AD	13-54	F	85	966	14	NA	15	VI	3
AD	03-30	F	60	1080	14.75	NA	14.75	VI	3
AD	12-27	F	89	1048	15	NA	15	VI	3
AD	00-06	M	72	NA	NA	5	NA	III	3
AD	00-13	M	78	NA	NA	5	NA	III	3
AD	00-19	M	80	NA	NA	5	NA	III	3
AD	00-23	F	64	NA	NA	5	NA	III	3
AD	00-24	M	73	NA	NA	5	NA	III	3
AD	00-31	M	79	NA	NA	5	NA	III	3
AD	00-39	M	80	NA	NA	5	NA	III	3
AD	00-71	F	88	1100	NA	5	NA	III	3
Mean ± 95% CI		6F/9M	79.27 ± 4.91	1056 ± 63	13.75 ± 0.99	5.00 ± 0.000	13.57 ± 1.980		3.00 ± 0.000
P value		0.2213	0.2335	0.0006	0.0076	0.0003	0.0006	<0.0001	<0.0001

[A] Statistics are as follows: Fisher's exact test for gender. [B] Unpaired two-tailed *t* test for age and brain weight. [C] Mann-Whitney test for plaque total, thal stage, tangle stage, and Braak score.

Supplemental Table 2. Antibodies and Reagents

Reagents	Dilution ratio	Source	Identifier
Rabbit Anti-ATP6V1A (polyclonal)	1:1000	Proteintech	CAT# 17115-1-AP
Rabbit Anti-ATP6V1B2 (polyclonal)	1:1000	Proteintech	CAT# 15097-1-AP
Rabbit Anti-ATP6V1C1 (polyclonal)	1:1000	Proteintech	CAT# 16054-1-AP
Rabbit Anti-ATP6V1D (polyclonal)	1:1000	Proteintech	CAT# 14920-1-AP
Rabbit Anti-ATP6V1E1 (polyclonal)	1:1000	Proteintech	CAT# 15280-1-AP
Rabbit Anti-ATP6V1F (polyclonal)	1:1000	Proteintech	CAT# 17725-1-AP
Rabbit Anti-ATP6V0a1 (polyclonal)	1:1000	Proteintech	CAT# 13828-1-AP
Rabbit Anti-ATP6V0c (polyclonal)	1:1000	St.Johns laboratory	CAT# STJ118778-100
Rabbit Anti-ATP6V0d1 (polyclonal)	1:1000	Proteintech	CAT# 18274-1-AP
Mouse Anti-Beta actin (monoclonal)	1:5000	Proteintech	CAT# 66009-1-Ig
Mouse Anti-Synaptophysin (monoclonal)	1:1000	Cell signaling technology	CAT# 12270
Rabbit Anti-Cathepsin B (monoclonal)	1:1000	Cell signaling technology	CAT# 31718
Rabbit Anti-Cathepsin D (monoclonal)	1:1000	Cell signaling technology	CAT# 74089
Rabbit Anti-Synaptagmin 1 (polyclonal)	1:1000	Synaptic System	CAT# 105 103BT
Rabbit Anti-CLCN3 (monoclonal)	1:1000	Cell signaling technology	CAT#13359
Mouse anti-MAP2 (monoclonal)	1:500	Invitrogen	CAT# MA5-12826
Rabbit anti-PSD95 (monoclonal)	1:200	Cell signaling technology	CAT# 3450S
Guinea pig-anti-vGlut1 (monoclonal)	1:700	Synaptic Systems	CAT# 135 304
DAPI	300 nM	Invitrogen	CAT# D1306
HRP-conjugated Goat Anti-Rabbit IgG(H+L)	1:3000	Proteintech	CAT# SA00001-2
HRP-conjugated Goat Anti-Mouse IgG(H+L)	1:3000	Proteintech	CAT# SA00001-1
Goat anti-Mouse IgG (H+L) Highly Cross-Adsorbed Secondary Antibody, Alexa Fluor™ Plus 488	1:400	Invitrogen	CAT# A32723
Goat anti-Rabbit IgG (H+L) Highly Cross-Adsorbed Secondary Antibody, Alexa Fluor™ 594	1:400	Invitrogen	CAT# A-11037
Goat anti-Guinea Pig IgG (H+L) Highly Cross-Adsorbed Secondary Antibody, Alexa Fluor™ 647	1:400	Invitrogen	CAT# A-21450
Concanamycin A		Sigma Aldrich	CAT# C9705
N-Acetyl-L-cysteine		Sigma Aldrich	CAT# A7250
Oligomycin A		TOCRIS Bioscience	CAT# 4110
Thapsigargin		Enzo	CAT# BML-PE180-0001
Ouabain		Sigma Aldrich	CAT# O0200000
Bafilomycin A1		Cell signaling technology	CAT# 54645
NADH		Sigma Aldrich	CAT# N4505
Phosphoenol pyruvate		Sigma Aldrich	CAT# 10108294001
Pyruvate kinase		Fisher Scientific	CAT# AAJ61373EXV
MgATP		Sigma Aldrich	CAT# A9187
Digitonin		Sigma Aldrich	CAT# 300410
Percoll		GE Healthcare	CAT# 17-0891-01
Acridine Orange		Thermo Fisher Scientific	CAT# A1301
NaH_2PO_4		Santa Cruz Biotechnology	CAT# sc251042
Cs-methanesulfonate		Sigma Aldrich	CAT# C1426
CsCl		Sigma Aldrich	CAT# C3309
QX-314		Sigma Aldrich	CAT# 552233
phosphocreatine		Sigma Aldrich	CAT# P1937
Na_2GTP		Sigma Aldrich	CAT# 371701
UAR-EMS Uranyl Acetate Replacement Stain		Electron Microscopy Sciences	CAT# 22405
